# Addressing the mean-variance relationship in spatially resolved transcriptomics data with *spoon*

**DOI:** 10.1101/2024.11.04.621867

**Published:** 2024-11-08

**Authors:** Kinnary Shah, Boyi Guo, Stephanie C. Hicks

**Affiliations:** 1 Department of Biostatistics, Johns Hopkins Bloomberg School of Public Health, Baltimore, MD, USA; 2 Department of Biomedical Engineering, Johns Hopkins School of Medicine, Baltimore, MD, USA; 3 Center for Computational Biology, Johns Hopkins University, Baltimore, MD, USA; 4 Malone Center for Engineering in Healthcare, Johns Hopkins University, MD, USA

**Keywords:** spatial transcriptomics, spatially variable gene, empirical Bayes, mean-variance bias, Gaussian process regression

## Abstract

An important task in the analysis of spatially resolved transcriptomics data is to identify spatially variable genes (SVGs), or genes that vary in a 2D space. Current approaches rank SVGs based on either *p*-values or an effect size, such as the proportion of spatial variance. However, previous work in the analysis of RNA-sequencing identified a technical bias, referred to as the “mean-variance relationship”, where highly expressed genes are more likely to have a higher variance. Here, we demonstrate the mean-variance relationship in spatial transcriptomics data. Furthermore, we propose *spoon*, a statistical framework using Empirical Bayes techniques to remove this bias, leading to more accurate prioritization of SVGs. We demonstrate the performance of *spoon* in both simulated and real spatial transcriptomics data. A software implementation of our method is available at https://bioconductor.org/packages/spoon.

## 1 Introduction

Advances in transcriptomics have led to profiling gene expression in a 2D space using spatially resolved transcriptomics (SRT) technologies [1]. These technologies have already led to novel biological insights across diverse application areas, including cancer [2], developmental biology [3, 4], and neurodegenerative disease [5, 6]. These emerging data types have also motivated new computational challenges, such as spatially-aware quality control to identify low-quality observations [7] and spatially-aware clustering to identify discrete spatial domains [8]. Another common data analysis task with these data is to perform feature selection by identifying a set of spatially variable genes (SVGs) [9–14]. The top SVGs are identified by ranking the genes based on some metric, such as *p*-values or an effect size like the proportion of spatial variance [10]. Accurately identifying SVGs is important because the top features are often used for downstream analyses, such as dimensionality reduction or unsupervised clustering [15–19].

Recently, Weber et al. [9] developed a computational method to identify SVGs based on a nearest-neighbor Gaussian process (NNGP) regression model [20]. In the paper, the authors identified an important relationship in SRT data. Specifically, they found a relationship between the estimated spatial variation and the overall expression, where genes that have higher overall expression are more likely to be more spatially variable. This phenomenon, known as the “mean-variance relationship”, is a well-documented technical bias in genomics [21–29]. As previously shown in other sequencing-based technologies, the reason for this bias is due to the preprocessing and normalization steps that are often applied to raw gene expression counts, or the number of unique molecular identifiers (UMIs) mapping to each gene. Specifically, Weber et al. [9] used normalized log_2_-transformed gene expression as input to the NNGP model. These preprocessing techniques are widely used in bulk RNA-seq, scRNA-seq, and SRT data, because these transformations are assumed to enable the use of statistical models based on Gaussian distributions, rather than less tractable count-based distributions [10, 28, 30–32].

However, previous work in the analysis of bulk and scRNA-seq data has also shown that because counts have unequal variances (or larger counts have larger standard deviations compared to smaller counts [33]) (Figure S1A), applying these log-transformations is problematic as it can overcorrect (or large logcounts can have a smaller standard deviation than small logcounts) (Figure S1B). In these settings, it is important to account for the mean-variance relationship. Another way to think about the mean-variance relationship is to describe it as heteroskedasticity [34] in the context of using linear models. In contrast, homoskedasticity, in the case of profiling gene expression, would be if all genes in a sample had the same variance. When applying statistical models that assume homoskedasticity in the data, if we ignore the mean-variance relationship, our results would produce inefficient estimators or even incorrect results [22, 35, 36]. For example, in differential expression analysis, ignoring the mean-variance relationship can produce false positive differentially expressed genes [25].

To address this technical bias in SRT data, here we introduce the *spoon* framework, which was inspired by the limma-voom method [33] developed for bulk RNA-seq data. In this way, the name *spoon* incorporates the concepts of both “spatial” and its origin in RNA-seq. Using real and simulated SRT data, we show that *spoon* is able to correct for the mean-variance relationship leading to more accurately prioritizing SVGs. A software implementation of our method is available as an R/Bioconductor package (https://bioconductor.org/packages/spoon).

## 2 Materials and Methods

### 2.1 An overview of the *spoon* model and methodological framework

The *spoon* model was inspired by the limma-voom method [33], which estimates the mean-variance relationship to obtain precision weights for each observation to be used as input into a linear regression model to identify differentially expressed genes with bulk RNA-sequencing data [26]. In *spoon*, we use a similar idea. First, we use Empirical Bayes techniques to estimate observation- and gene-level weights. However, here we use a Gaussian process regression model, rather than a linear regression model, to model SRT data. Then, we leverage the Delta method to re-scale the data and covariates by these weights to address the heteroskedasticity in SRT data. Briefly, the Gaussian process (GP) regression model is specified as follows [20]:

(1) $$y(s)=x(s{)}^{\prime }\beta +w(s)+\epsilon (s)$$

where s are the spatial locations, y(s) is the response at a location, x(s) is a vector of explanatory variables, w(s) is a function accounting for the spatial dependence, and $\epsilon (s)∼N\left(0,\tau^{2}\right)$ is noise. β is a fixed effect, while w and ϵ are random effects. w(s) is modeled with a Gaussian process, w(s)∼GP(μ(s),C(θ)), where μ(s) is a mean function and C(θ) is a covariance function with parameters $\theta =\left(\sigma^{2},\;\phi ,\ldots \right)$ for the Matérn covariance function:

$$C\left(s_{i},s_{j}|\theta =\left(\sigma^{2},\phi ,\nu \right)\right)=\frac{\sigma^{2}}{2^{\nu -1}\Gamma (\nu )}{\left(\left\|s_{i}-s_{j}\right\|\phi \right)}^{\nu }K_{\nu }\left(\left\|s_{i}-s_{j}\right\|\phi \right);\;\phi >0,\;\nu >0$$

where $\sigma^{2}$ is the spatial component of variance, ϕ is the decay in spatial correlation, ν is the smoothness parameter, and $K_{\nu }$ is the Bessel function of the second kind with order ν. Because we fit these models on a per-gene basis with up to thousands of genes in a given dataset, we use a nearest-neighbor Gaussian process (NNGP) [37, 38] to reduce the computational running time and make *spoon* useful to practitioners. The key idea behind using NNGPs is that instead of conditioning on all of the points in the data, only a subset (a set of nearest neighbors) of the data are used for the conditioning. Conditioning on enough of the closest neighbors provides sufficient estimates of the needed information needed and improves storage and computational costs. Briefly, a NNGP is fit to the preprocessed expression values for each gene:

(2) $$y∼N(X\beta ,\tilde{\Sigma }\left(\theta ,\tau^{2}\right))$$

where the primary difference between a full GP model (Equation 1) and a NNGP (Equation 2) is that the NNGP covariance matrix, $\tilde{\Sigma }\left(\theta ,\tau^{2}\right)$, is a computationally fast approximation to the covariance matrix from a full GP model, $\Sigma \left(\theta ,\tau^{2}\right)=C(\theta )+\tau^{2}I$. In other words, $\tilde{\Sigma }$ approximates the covariances from both from w(s) and ϵ(s). For the kernel, $C(\theta )=\left[C_{ij}(\theta )\right]$, we assume an exponential covariance function:

$$C_{ij}(\theta )=\sigma^{2}exp\left(\frac{-\left\|s_{i}\;-\;s_{j}\right\|}{l}\right)$$

where $\theta =\left(\sigma^{2},\;l\right)$, and $\sigma^{2}$ is the spatial component of variance of interest. $\sigma^{2}$ is different from the nonspatial component of variance, $\tau^{2}$, which is also referred to as the nugget. l is the lengthscale parameter, which sets how quickly the correlation decays with distance. $\left\|s_{i}-s_{j}\right\|$ is the Euclidean distance between spatial locations. To estimate the parameters in the NNGP model, we use the BRISC R package [20]. Using the estimated parameters, we calculate an effect size, the proportion of spatial variance $\left(\frac{{\overset{ˆ}{\sigma }}^{2}}{{\overset{ˆ}{\sigma }}^{2}+{\overset{ˆ}{\tau }}^{2}}\right)$.

### 2.2 Calculating observation- and gene-level weights using Empirical Bayes techniques

**Figure 1:**
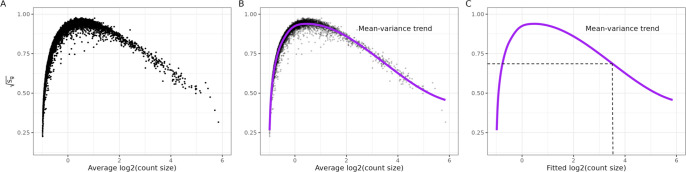
Calculating precision weights for individual observations. These data are from Invasive Ductal Carcinoma breast tissue analyzed with 10x Genomics Visium [39], hereafter referred to as “Ductal Breast”. (**A-C**) The square root of the residual standard deviations estimated using nearest neighbor Gaussian processes ($\sqrt{s_{g}}$ defined in Equation 3) are plotted against average logcount ($\tilde{r}$). (**B**) Same as A, except a spline curve (purple) is fitted to the data to estimate the gene-wise mean-variance relationship. (**C**) Using the fitted spline curve, each predicted count value $\left({\overset{ˆ}{\lambda }}_{gi}\right)$ is mapped to its corresponding square root standard deviation value using $spl{\left({\overset{ˆ}{\lambda }}_{gi}\right)}^{-4}$.

Briefly, we calculate the average log_2_ expression values and the standard deviations of the residuals from fitting an NNGP model per gene using BRISC (Figure 1A). Then, we use splines to fit the gene-wise mean-variance relationship (Figure 1B). Finally, we use the fitted curve to estimate observation- and gene-level weights (Figure 1C). Next, we describe each of these steps in greater detail.

#### 2.2.1 Fitting per-gene NNGP models using logCPM values

We start with a counts matrix, transposed so each row is a spot and each column is a gene. There are n spots and G genes in the counts matrix. The UMI counts can be indexed by $r_{gi}$ for spots i=1 to n and genes g=1 to G. We define the total number of UMIs for sample i as $R_{i}=\sum_{g=1}^{G}\;r_{gi}$. Next, we transform $r_{gi}$ to adjust for the total number of UMIs $\left(R_{i}\right)$ by using logcounts per million (logCPM). We use a pseudocount of 0.5 to ensure we do not take the log of 0 and we add a pseudocount of 1 to the library size to make sure $0<\frac{\left(r_{gi}+0.5\right)}{R_{i}+1}<1$:

$$y_{gi}={log}_{2}\left(\frac{r_{gi}\;+\;0.5}{R_{i}\;+\;1}\times 10^{6}\right)$$

Using the normalized and ${log}_{2}$-transformed data $y_{gi}$, we fit a NNGP model (Equation 2) per gene with a default of $X=1_{\left[N\times 1\right]}$, corresponding to including an intercept, with $\beta_{g}$ representing the overall mean expression level for gene g. Using the observed data $y_{gi}$ and the predicted value ${\overset{ˆ}{\mu }}_{gi}=x_{i}^{T}{\overset{ˆ}{\beta }}_{g}$, we can calculate the standard deviation of the residuals between $y_{gi}$ and ${\overset{ˆ}{\mu }}_{gi}$:

(3) $$s_{g}=\sqrt{\frac{\sum_{i=1}^{n}\;{\left(y_{gi}\;-\;{\overset{ˆ}{\mu }}_{gi}\right)}^{2}}{n\;-\;1}}$$

The square root of $s_{g}$ is what we use to represent the ‘variance’ in the mean-variance relationship (see *y*-axis in Figure 1A–C). This concept is used in limma-voom as well because the square root of the standard deviations is roughly symmetrically distributed.

#### 2.2.2 Modeling the mean-variance relationship using $\sqrt{s_{g}}$ and $\tilde{r}$

Next, we fit a nonparametric spline curve to model the mean-variance relationship in our data. Instead of using ${\overset{‾}{y}}_{g}$ directly to represent the ‘mean’ component, we convert ${\overset{‾}{y}}_{g}$ to average logcount using the geometric mean of library size, $\tilde{R}=exp\left(\sum_{i=1}^{n}\;log\left(R_{i}\right)\right)$. We use the geometric mean to avoid integer overflow:

(4) $$\tilde{r}={\overset{‾}{y}}_{g}+{log}_{2}(\tilde{R})-{log}_{2}\left(10^{6}\right)$$

Then, we use smoothing splines (specifically smooth.spline() in the base R stats package) to model the mean-variance relationship between $\sqrt{s_{g}}$ and $\tilde{r}$. We use splines because we found they are a robust way to model the mean variance relationship seen across multiple datasets. We use the notation spl() to denote the fitted curve (Figure 1B), which represents an estimate of the mean-variance relationship.

#### 2.2.3 Prediction modeling using fitted spl() curve

Similar to Equation 4, we convert the predicted value ${\overset{ˆ}{\mu }}_{gi}$ (on the logCPM scale) to a predicted count value:

(5) $${\overset{ˆ}{\lambda }}_{gi}={\overset{ˆ}{\mu }}_{gi}+{log}_{2}\left(R_{i}+1\right)-{log}_{2}\left(10^{6}\right)$$

The fitted counts values for each observation are used as input to predict the square root residual standard deviation values for each $y_{gi}$ using the spline curve. Figure 1C shows an example of mapping an individual observation to a square-root standard deviation value using its fitted value from the BRISC models.

To avoid extrapolating beyond the range of the function, individual observations that have ${\overset{ˆ}{\lambda }}_{gi}$ more extreme than the range of $\tilde{r}$ are constrained. If ${\overset{ˆ}{\lambda }}_{gi}$ is greater than $max(\bar{y})$, then the predicted square root residual standard deviation value for that observation is constrained to $spl(max(\bar{y}))$. If ${\overset{ˆ}{\lambda }}_{gi}$ is less than $min(\bar{y})$, then the predicted square root residual standard deviation value for that observation is constrained to $spl(min(\bar{y}))$. The final step is taking the inverse of the squared predicted standard deviation to compute the weight for each individual observation. The weight for each observation is defined as $w_{gi}=spl\;{\left({\overset{ˆ}{\lambda }}_{gi}\right)}^{-4}$, using the constrained values for observations outside of the range.

### 2.3 Correct for heteroskedasticity using observation- and gene-level precision weights

If the desired SVG detection method accepts observation- and gene-level weights, then the estimated weights $w_{gi}$ (described in Section 2.2) can be used as input directly into the method. If the desired SVG detection method does not accept weights, then the Delta method is leveraged to rescale the data and covariates by the weights. These scaled data and covariates are used as inputs into the desired SVG detection function.

For example, the SVG detection tool called nearest neighbor SVGs (nnSVG) [9] uses a Gaussian process regression model and can have weights incorporated in the following way. We correct for the heteroskedasticity by adjusting with precision weights, $w_{gi}$ for gene g at spatial location i. If W is a diagonal matrix where each diagonal element is $w_{gi}$, then we know:

Wy∼N(WXβ,WΣW)

where

$$W\Sigma W=WC(\theta )W+\tau^{2}WIW\;\;=WC(\theta )W+\tau^{2}WW\;\;=C\left(\theta^{\prime }\right)+\tau^{\prime 2}I$$

and the new input data to nnSVG would be Wy and WX where $X=\left[\begin{array}{c} 1\\ X_{gi} \end{array}\right]$.

### 2.4 Data

#### 2.4.1 Real SRT data

Tissues from several regions of the human body analyzed with 10x Genomics Visium were used in the analyses. The datasets and preprocessing steps are further described below:

- Ductal Breast: Invasive Ductal Carcinoma breast tissue data are publicly available from the 10x Genomics website. It contains one tissue sample from one donor with Invasive Ductal Carcinoma [39]. After preprocessing, this dataset contains 12,321 genes and 4,898 spots.
- Lobular Breast: Invasive Lobular Carcinoma breast tissue data are publicly available from the 10x Genomics website. It contains one tissue sample from one donor with Invasive Lobular Carcinoma [40]. After preprocessing, this dataset contains 12,624 genes and 4,325 spots.
- Subtype Breast: Estrogen receptor positive (ER+) breast cancer tissue data are publicly available on Zenodo and contains several tissue samples of breast cancer tissue. Only sample CID4290 is used for this analysis [41]. After preprocessing, this dataset contains 12,325 genes and 2,419 spots.
- DLPFC: This dataset contains two pairs of spatial replicates of human postmortem dorsolateral prefrontal cortex (DLPFC) tissue from three neurotypical adult donors. Only tissue sample 151507 is used for this analysis [15]. After preprocessing, this dataset contains 7,343 genes and 4,221 spots.
- HPC: This dataset contains human postmortem hippocampus (HPC) tissue from several neurotypical adult donors. Each sample was broken up into four Visium slides due to the large size of the HPC. Only tissue sample V12D07_335, portion D1 is used for this analysis [16]. After preprocessing, this dataset contains 5,348 genes and 4,992 spots.
- LC: This dataset contains human postmortem locus coeruleus (LC) tissue from five neurotypical adult donors. Only tissue sample 2701 is used for this analysis [42]. After preprocessing, this dataset contains 1,331 genes and 2,809 spots.
- Ovarian: This dataset contains tissues collected during interval debulking surgery from eight high-grade serous ovarian carcinoma patients undergoing chemotherapy. Only one tissue sample from patient 2 is used for this analysis [43]. After preprocessing, this dataset contains 12,022 genes and 1,935 spots.

Preprocessing was performed as uniformly as possible across the datasets. For datasets that had an annotation for whether or not a spot was in the tissue, spots outside of the tissue were removed. For the Subtype Breast dataset, spots that were classified as artifacts were removed. nnSVG::filter_genes() was used to remove genes without enough data, specifically we kept genes with at least 2 counts in at least 0.2% of spots. For the LC dataset, we used a UMI filter instead of this function to remove genes with less than 80 total UMI counts summed across all spots. scuttle::logNormCounts() with default arguments was used to compute log-normalized expression values.

#### 2.4.2 Simulated SRT data

To simulate the mean-variance relationship, we simulated raw gene expression counts following a Poisson distribution:

$$c\left(s\right)|\lambda \left(s\right)∼Poisson\left(\lambda \left(s\right)\right);\lambda (s)=exp\left(\beta +C\left(\sigma^{2}\right)\right)$$

where s are spatial locations, β is a vector of true mean expression per gene, $\sigma^{2}$ is the spatial component of variance, and C is the covariance function using a Matérn kernel with squared exponential distance. The $\sigma^{2}$ values and β values were randomly assigned from ranges of [0.2,1] and [ln(0.5),ln(1)], respectively. We intentionally simulate $\sigma^{2}$ and β values so they are not correlated. In this way, we ensure we are simulating SVGs at all levels of mean expression. A fixed lengthscale parameter was chosen for all of the genes in a given simulation. Based on the estimated lengthscale distributions for four datasets, we chose to focus our simulations on smaller lengthscales because the majority of estimated lengthscales are between 0 to 0.15 (Figure S2). For reference, a scaled lengthscale value of 0.15 is interpreted as 15% of the maximum width or height of the tissue area on a standard Visium slide. We simulated 1000 genes in the following simulations.

In addition, we also considered the performance as a function of varying the lengthscale parameter l in $\theta =\left(\sigma^{2},\;l\right)$. In the NNGP model, the lengthscale parameter sets how quickly the correlation decays with distance. In the nnSVG SVG detection method [9], a key innovation was using a flexible lengthscale parameter to fit the model for each gene. Genes within the same tissue can spatially vary with different ranges of sizes and patterns, so a flexible lengthscale parameter for each gene enables the discovery of distinct biological processes. For the primary simulation evaluation, a lengthscale of 100 was used. This corresponds to a scaled lengthscale value of roughly 0.02. For supplementary simulation evaluations, 50, 60, 100, and 500 lengthscales were used. These correspond to 0.010, 0.012, 0.020, and 0.100 of the maximum width or height of the tissue area on a standard Visium slide. The spatial coordinates from the example dataset Visium_DLPFC() in the STexampleData package were used. This dataset contains 4,992 spots. We used the subset of 968 spots with row and column coordinates between 20 to 65 as the spatial coordinates to reduce the amount of time to simulate data.

### 2.5 Methods to detect SVGs

For Moran’s I [44], we ranked genes by the Moran’s I value. For nnSVG [9], the genes were ranked within the method based on the estimated likelihood ratio test statistic values comparing the fitted model against a classical linear model, assuming the spatial component of variance is zero. For SpaGFT [45], the gene ranks were calculated within the method based on decreasing GFTscore, a measure of randomness of gene expression. For SPARK-X [11], adjusted combined *p*-values from multiple covariance matrices and kernels were used to rank genes. For SpatialDE2 [46], the genes were ranked by the negative of the fraction of spatial variance for each gene. All of the criteria were ranked using the ties.method = “first” option.

1. Moran’s I: Rfast2::moranI() [47] was used to compute Moran’s I values, and the negative Moran’s I value for each gene was ranked.
2. nnSVG: nnSVG::nnSVG() [9] was used, and the rank was calculated as part of the output of the function.
3. SpaGFT: SpaGFT.detect_svg() [45] was implemented in Python, and the rank was calculated as part of the output of the function.
4. SPARK-X: SPARK::sparkx() [11] was run with the option of a mixture of various kernels. The combined *p*-value from all the kernels for each gene was ranked.
5. SpatialDE2: SpatialDE.fit() [46] was implemented in Python to fit the model for each gene. The negative of the fraction of spatial variance for each gene was ranked.

An intercept-less covariate matrix is required to implement a weighted version of an SVG detection method. To the best of our knowledge, nnSVG is the only SVG detection tool with the option to include a covariate matrix without an intercept term. The weights from *spoon* have the potential to integrate with other methods based on the flexibility of their design.

### 2.6 Code Availability

*spoon* is freely available for use as an R package available from Bioconductor at https://bioconductor.org/packages/spoon. The code to reproduce the analyses in this paper is available on GitHub at https://github.com/kinnaryshah/MeanVarBias. We used *spoon* version 1.1.3 and R version 4.4.1 for the analyses in this manuscript.

## 3 Results

### 3.1 The mean-variance relationship exists in spatial transcriptomics data

**Figure 2:**
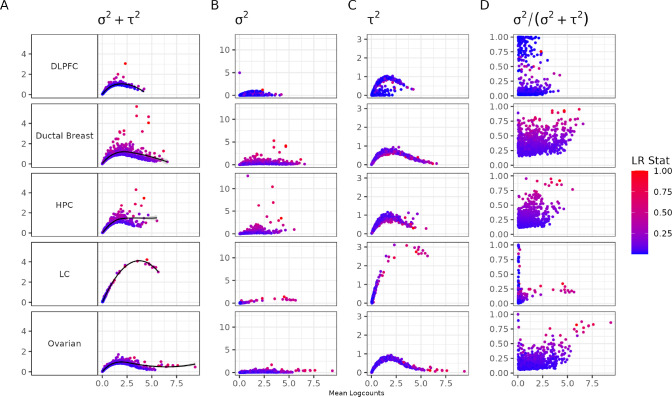
Mean-variance relationship exists in spatially resolved transcriptomics. Using data from different human tissues, in order from top to bottom: DLPFC [15], Ductal Breast cancer [39], HPC [16], LC [42], and Ovarian cancer [43], we quantified the mean-variance relationship. Each point is a gene colored by the likelihood ratio statistic for a test that compares the fitted model against a classical linear model for the spatial component of variance using a NNGP [9]. The likelihood ratio statistics (LR Stat) are scaled by the maximum likelihood ratio statistic for each dataset in order to have more uniform visualization. The x-axis is mean logcounts and the y-axes represent different components of variance, in order from left to right: total variance $\sigma^{2}+\tau^{2}$, spatial variance $\sigma^{2}$, nonspatial variance $\tau^{2}$, and proportion of spatial variance $\sigma^{2}/\left(\sigma^{2}+\tau^{2}\right)$.

We begin by systematically demonstrating the mean-variance relationship in SRT data. This finding builds upon the initial finding suggested in Weber et al. [9]. In contrast to investigating this bias in one tissue from one tissue section, here we explore this finding across multiple tissue sections from different regions in the human body, namely DLPFC, Ductal Breast cancer, HPC, LC, and Ovarian cancer. To visualize the mean-variance relationship, we plot the mean logcounts against different components (spatial and non-spatial components) of variance calculated using nnSVG. As seen in Figure 2, the mean-variance relationship is a concern in SRT data, specifically in the nonspatial component of variance, $\tau^{2}$. Given $\tau^{2}$ is used when calculating the proportion of spatial variance, this suggests the way genes are prioritized as spatially variable is dependent on the overall mean expression for the gene.

Next, we further investigated one of these tissues (DLPFC) to ask if the mean-variance relationship was due to differences in the spatial domains of the tissue. The six layers in the human neocortex are transcriptionally quite different from one another [15], so we wanted to show that the mean-variance relationship still exists when stratifying by layers. In order to control for differences in layer domains, the DLPFC data was first separated into Layers I-VI, and white matter and then the mean logcounts were plotted against the components of variance for each layer in the brain. However, we found that the mean-variance relationship was still observed within the different biological domains (Figure S3).

### 3.2 The mean-rank relationship exists in other SVG detection methods

Having established that the mean-variance relationship exists in SRT data across different tissues as measured by Gaussian processes in nnSVG, we next explored the mean-rank relationship as an extension of the mean-variance relationship. Other SVG detection methods do not separate out the total variance into spatial and nonspatial variance components, so we examine the mean-variance relationship using this proxy.

**Figure 3:**
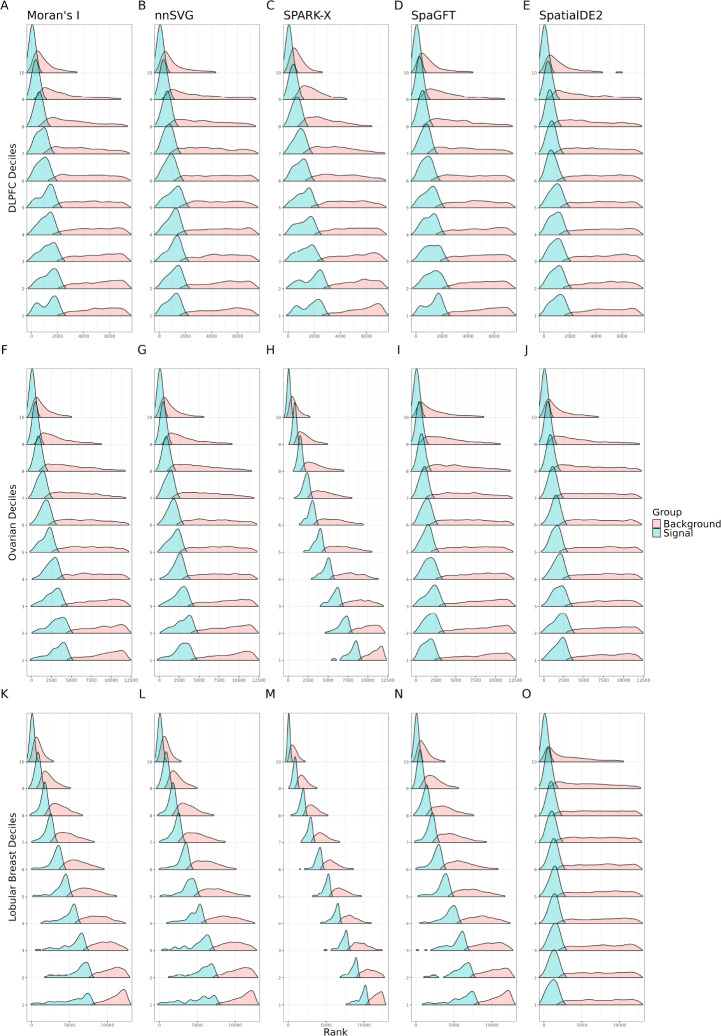
Mean-rank relationship exists in spatial transcriptomics data. Using three datasets, in order from top to bottom (DLPFC [15], Ovarian cancer [39], and Lobular Breast cancer [40]), we quantified the mean-rank relationship. The genes were binned into deciles based on mean logcounts. Decile 1 contains the lowest mean expression values. The x-axis represents the rank. Within each decile, the density of the top 10% ranks is plotted as the signal in blue, while the density of the remaining ranks is plotted as the background in orange. Each subfigure shows the mean-rank relationship that persists after applying each method, from left to right: Moran’s I [47], nnSVG [9], SPARK-X [11], SpaGFT [45], and SpatialDE2 [46].

We examined the mean-rank relationship from several popular SVG detection methods on the DLPFC, Ovarian cancer, and Lobular Breast cancer datasets (Figure 3). The ranks were calculated for each SVG method (described in Section 2.5). We found that for almost every method, there is a clear relationship between the mean and the rank. Stated another way, the SVG detection methods that we evaluated rank and prioritize genes as SVGs, which is related to the overall mean expression. Because the overall mean expression is likely a technical artifact, we would expect that there should be genes that are highly ranked as SVGs within each mean-level decile. However, what we found is that the mean-variance relationship biases genes towards the higher mean expression deciles. The extreme bias observed in SPARK-X is also noted in a recent benchmarking paper [48]. These are state of the art methods that perform well in recent benchmarking papers [14, 48, 49], yet they are sorely affected by the mean-variance bias.

### 3.3 Simulation: Weighted Spatially Variable Gene Evaluation

**Figure 4:**
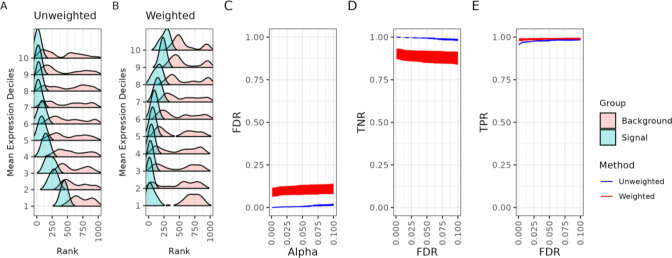
*Spoon* removes the mean-variance relationship when detecting spatially variable genes. This dataset consists of 1,000 simulated genes across 968 spots using a lengthscale of 100. Separately for unweighted and weighted methods, the genes were binned into deciles based on mean logcounts. Decile 1 contains the lowest mean expression values. Ridge plots for the (**A**) unweighted ranks and (**B**) weighted ranks are shown. Within each decile (*y*-axis), the density of the top 10% of ranks is plotted as the signal, while the density of the remaining ranks is plotted as the background. (**C**) False discovery rate (FDR) as a function of Type I error (α). As a function of FDR, we show the (**D**) true negative rate (TNR) and (**E**) true positive rate (TPR). The red represents weighted nnSVG and the blue represents unweighted nnSVG. These plots represent the average performance across five iterations of the same simulation, each with unique random seeds.

To address the mean-variance and mean-rank relationships, we began with simulation studies to evaluate the performance of *spoon* under different scenarios. Using simulated raw gene expression counts following a Poisson distribution (Section 2.4.2) with a fixed lengthscale (l=100), we ranked SVGs using nnSVG [9] without weights and with weights estimated via *spoon*. We found a strong mean-rank relationship using the unweighted SVGs (Figure 4A) compared to the weighted SVGs using *spoon* (Figure 4B). Stated differently, using observational- and gene-level weights, we can identify highly ranked SVGs even in lower deciles, demonstrating that *spoon* effectively addresses the mean-variance relationship.

We also explored the false discovery rate (FDR) (Figure 4C), true negative rate (TNR) (Figure 4D), and true positive rate (TPR) (Figure 4E). The red represents weighted nnSVG and the blue represents unweighted nnSVG. These plots represent the average of each respective rate over five iterations of the same simulation with unique random seeds. The FDR and TNR are similar between the unweighted and weighted methods, with a slight increase in performance observed in the unweighted method. The TPR, however, is very similar for both methods. Finally, we considered other lengthscale values and found that the mean-variance relationship is improved for all values tested (Figure S4). We found that the weights from *spoon* improve the TPR for smaller lengthscale values, and there are diminishing returns regarding the convergence of the TPR for both the weighted method and unweighted methods at larger lengthscale values.

### 3.4 Real Data: Weighted Spatially Variable Gene Evaluation

**Figure 5:**
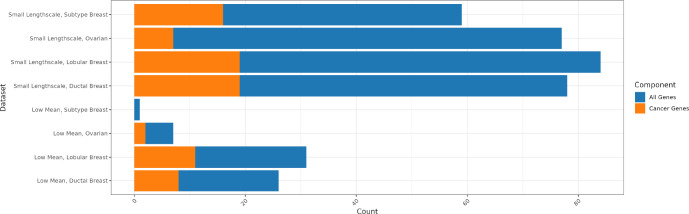
*Spoon* helps to detect SVGs associated with cancer that are lowly expressed. We used four datasets to evaluate the detection of cancer-related genes: Subtype Breast cancer [41], Ovarian cancer [43], Lobular Breast cancer [40], and Ductal Breast cancer [39]. Each bar contains the intersection of the set of genes of interest with genes within the set associated with cancer. For the first four rows, we defined low mean genes as those with means less than the 25th percentile in the dataset. Within the set of low mean genes, we found genes that were in the lowest 10% of ranks before weighting and then increased to the highest 10% of ranks after weighting. This is the set of genes of interest. The intersection in orange is the number of low mean and higher ranked genes that were found to be associated with the cancer of the dataset. For the last four rows, we defined small lengthscale genes as those with lengthscales between 40 to 90. Within the set of small lengthscale genes, we found genes that were ranked higher after weighting. This is the set of genes of interest. The intersection in orange shows the number of small lengthscale genes that were ranked higher and found to be associated with the cancer type of the dataset.

Next, we evaluated the downstream impact of incorporating weights from *spoon* into SVG detection methods. Here, we aimed to demonstrate the impact of our method on recovering lowly expressed genes that become highly ranked in real biological datasets. We defined small mean gene expression genes as those with means less than the 25th percentile in the dataset. Within the set of small mean gene expression, we identified genes that were in the lowest 10% of ranks before weighting and then increased to the highest 10% of ranks after weighting. In the Ovarian cancer dataset, there are 7 genes that met this criterion. Out of these 7 genes, *TUFT1* and *DDX39B* are known to be implicated in ovarian cancer [50, 51]. These potentially important SVGs were ignored due to their low expression levels and our weighting algorithm can recapture them. Similar analyses were performed for the other three cancer datasets (Figure 5). The gene lists can be found in the supplemental materials.

Then, we explored the improvement in the small lengthscale set of genes. We defined small lengthscale genes as those with lengthscale values between 40 to 90. Within the set of small lengthscale genes, we found genes that were ranked higher after weighting. We also derived the “null distribution” — the underlying total SVGs for each dataset as a point of reference for the proportion of small lengthscale genes that are ranked higher. We found that the differing proportions of small lengthscale genes that become higher ranked after weighting is appropriate based on the “null distribution” of the proportion of unweighted SVGs (Figure S5). Again, we related the higher-ranked small lengthscale genes to the cancer type of the dataset. In the Subtype Breast dataset, 59 small lengthscale genes were higher ranked after weighting, with 16 of these genes implicated in breast cancer. Full results are presented in Figure 5 and gene lists are in supplemental materials.

## 4 Discussion

In our work, we systematically demonstrate the mean-variance and the mean-rank relationships exist in spatially resolved transcriptomics data. Furthermore, we show this is not limited to just one SVG detection method. If researchers fail to adjust for this bias in spatial transcriptomics data, this can lead to false positives and inaccurate rankings of SVGs due to the violation of the homoskedasticity assumption. Here, we show that our method *spoon* is able to correct for this bias. Specifically, our approach uses Empirical Bayes techniques to generate weights for downstream analyses to remove the mean-variance relationship, leading to a more informative set of SVGs.

In a recent benchmark evaluation of SVG detection methods, the authors Chen et al. [48] noted a similar bias. NoVaTeST was recently proposed as a method to identify SVGs allowing noise variance to vary with spatial locations [52]. This method aims to identify genes that have location-dependent noise variance in SRT data, or genes that have statistically significant heteroskedasticity. This noise variation can be due to technical noise from the mean-variance relationship, variation due to sequencing processes, or underlying biological differences, making it difficult to parse out the mean-variance relationship. Additionally, further analysis of the genes detected by NoVaTest showed that some genes are likely affected by the mean-variance relationship, and the authors suggest using a strong variance-stabilizing transformation.

We recognize there are limitations to our project and aim to address these in future work. Primarily, simulation studies for spatial transcriptomics data are difficult to design and execute due to numerical instability and limitations of parameterization. There is no clear consensus on the definition of an SVG, so we chose to simulate overall SVGs, defined in Yan et al. [53] as genes that exhibit non-random spatial patterns. To our knowledge, we are not aware of methods to simulate SVGs that include the mean-variance bias. In future work, we aim to refine spatial transcriptomics simulation study design to incorporate the mean-variance relationship and have more flexibility with various parameters, such as mean gene expression, degree of spatial variation, expression strength, and varying effect sizes in the same simulated dataset. We found that our method is most powerful for small lengthscale genes, and we hope to better understand medium and large lengthscale genes in future work as well.

In sum, we provide evidence for the mean-variance and mean-rank relationship in SRT data and show that our method *spoon* can mitigate these biases. We offer the software as an easily installable R/Bioconductor package that interfaces with SpatialExperiment to make this method broadly accessible to researchers.

## Supplementary Material

Supplement 1

## References

[1] Marx V.. Method of the Year: spatially resolved transcriptomics. Nature Methods, 18(1):9–14, Jan. 2021. ISSN 1548–7091, 1548–7105. doi:10.1038/s41592-020-01033-y. URL https://www.nature.com/articles/s41592-020-01033-y. 33408395

[2] Deshpande A., Loth M., Sidiropoulos D. N., Zhang S., Yuan L., Bell A. T., Zhu Q., Ho W. J., Santa-Maria C., Gilkes D. M., Williams S. R., Uytingco C. R., Chew J., Hartnett A., Bent Z. W., Favorov A. V., Popel A. S., Yarchoan M., Kiemen A., Wu P.-H., Fujikura K., Wirtz D., Wood L. D., Zheng L., Jaffee E. M., Anders R. A., Danilova L., Stein-O’Brien G., Kagohara L. T., and Fertig E. J.. Uncovering the spatial landscape of molecular interactions within the tumor microenvironment through latent spaces. Cell Systems, 14(4):285–301.e4, Apr. 2023. ISSN 24054712. doi:10.1016/j.cels.2023.03.004. URL https://linkinghub.elsevier.com/retrieve/pii/S2405471223000807. 37080163 PMC10236356

[3] Rao A., Barkley D., França G. S., and Yanai I.. Exploring tissue architecture using spatial transcriptomics. Nature, 596(7871):211–220, Aug. 2021. ISSN 0028–0836, 1476–4687. doi:10.1038/s41586-021-03634-9. URL https://www.nature.com/articles/s41586-021-03634-9. 34381231 PMC8475179

[4] Garcia-Alonso L., Lorenzi V., Mazzeo C. I., Alves-Lopes J. P., Roberts K., Sancho-Serra C., Engelbert J., Marečková M., Gruhn W. H., Botting R. A., Li T., Crespo B., Van Dongen S., Kiselev V. Y., Prigmore E., Herbert M., Moffett A., Chédotal A., Bayraktar O. A., Surani A., Haniffa M., and Vento-Tormo R.. Single-cell roadmap of human gonadal development. Nature, 607(7919):540–547, July 2022. ISSN 0028–0836, 1476–4687. doi:10.1038/s41586-022-04918-4. URL https://www.nature.com/articles/s41586-022-04918-4. 35794482 PMC9300467

[5] Chen K. S., Noureldein M. H., Rigan D. M., Hayes J. M., Savelieff M. G., and Feldman E. L.. Regional interneuron transcriptional changes reveal pathologic markers of disease progression in a mouse model of Alzheimer’s disease, Nov. 2023. URL 10.1101/2023.11.01.565165v1.

[6] Vanrobaeys Y., Peterson Z. J., Walsh E. N., Chatterjee S., Lin L.-C., Lyons L. C., Nickl-Jockschat T., and Abel T.. Spatial transcriptomics reveals unique gene expression changes in different brain regions after sleep deprivation. Nature Communications, 14(1):7095, Nov. 2023. ISSN 2041–1723. doi:10.1038/s41467-023-42751-z. URL https://www.nature.com/articles/s41467-023-42751-z. PMC10625558 37925446

[7] Totty M., Hicks S. C., and Guo B.. SpotSweeper: spatially-aware quality control for spatial transcriptomics, June 2024. URL 10.1101/2024.06.06.597765. PMC12258134 40481362

[8] Yuan Z., Zhao F., Lin S., Zhao Y., Yao J., Cui Y., Zhang X.-Y., and Zhao Y.. Benchmarking spatial clustering methods with spatially resolved transcriptomics data. Nature Methods, 21(4):712–722, Apr. 2024. ISSN 1548–7091, 1548–7105. doi:10.1038/s41592-024-02215-8. URL https://www.nature.com/articles/s41592-024-02215-8. 38491270

[9] Weber L. M., Saha A., Datta A., Hansen K. D., and Hicks S. C.. nnSVG for the scalable identification of spatially variable genes using nearest-neighbor Gaussian processes. Nature Communications, 14 (1):4059, July 2023. ISSN 2041–1723. doi:10.1038/s41467-023-39748-z. URL https://www.nature.com/articles/s41467-023-39748-z. PMC10333391 37429865

[10] Svensson V., Teichmann S. A., and Stegle O.. SpatialDE: identification of spatially variable genes. Nature Methods, 15(5):343–346, May 2018. ISSN 1548–7091, 1548–7105. doi:10.1038/nmeth.4636. URL https://www.nature.com/articles/nmeth.4636. 29553579 PMC6350895

[11] Zhu J., Sun S., and Zhou X.. SPARK-X: non-parametric modeling enables scalable and robust detection of spatial expression patterns for large spatial transcriptomic studies. Genome Biology, 22(1):184, Dec. 2021. ISSN 1474–760X. doi:10.1186/s13059-021-02404-0. URL 10.1186/s13059-021-02404-0. 34154649 PMC8218388

[12] Hao M., Hua K., and Zhang X.. SOMDE: a scalable method for identifying spatially variable genes with self-organizing map. Bioinformatics, 37(23):4392–4398, Dec. 2021. ISSN 1367–4803, 1367–4811. doi:10.1093/bioinformatics/btab471. URL https://academic.oup.com/bioinformatics/article/37/23/4392/6308937. 34165490

[13] Dries R., Zhu Q., Dong R., Eng C.-H. L., Li H., Liu K., Fu Y., Zhao T., Sarkar A., Bao F., George R. E., Pierson N., Cai L., and Yuan G.-C.. Giotto: a toolbox for integrative analysis and visualization of spatial expression data. Genome Biology, 22(1):78, Dec. 2021. ISSN 1474–760X. doi:10.1186/s13059-021-02286-2. URL 10.1186/s13059-021-02286-2. 33685491 PMC7938609

[14] LiZ., PatelZ. M., SongD., YanG., LiJ. J., and PinelloL.. Benchmarking computational methods to identify spatially variable genes and peaks, Dec. 2023. URL 10.1101/2023.12.02.569717.40968359

[15] Maynard K. R., Collado-Torres L., Weber L. M., Uytingco C., Barry B. K., Williams S. R., Catallini J. L., Tran M. N., Besich Z., Tippani M., Chew J., Yin Y., Kleinman J. E., Hyde T. M., Rao N., Hicks S. C., Martinowich K., and Jaffe A. E.. Transcriptome-scale spatial gene expression in the human dorsolateral prefrontal cortex. Nature Neuroscience, 24(3):425–436, Mar. 2021. ISSN 1546–1726. doi:10.1038/s41593-020-00787-0. URL https://www.nature.com/articles/s41593-020-00787-0. 33558695 PMC8095368

[16] Nelson E. D., Tippani M., Ramnauth A. D., Divecha H. R., Miller R. A., Eagles N. J., Pattie E. A., Kwon S. H., Bach S. V., Kaipa U. M., Yao J., Kleinman J. E., Collado-Torres L., Han S., Maynard K. R., Hyde T. M., Martinowich K., Page S. C., and Hicks S. C.. An integrated single-nucleus and spatial transcriptomics atlas reveals the molecular landscape of the human hippocampus, Apr. 2024. URL http://biorxiv.org/lookup/doi/10.1101/2024.04.26.590643. 10.1038/s41593-025-02022-0 PMC12411265 40739059

[17] Walker B. L., Cang Z., Ren H., Bourgain-Chang E., and Nie Q.. Deciphering tissue structure and function using spatial transcriptomics. Communications Biology, 5(1):1–10, Mar. 2022. ISSN 2399–3642. doi:10.1038/s42003-022-03175-5. URL https://www.nature.com/articles/s42003-022-03175-5. 35273328 PMC8913632

[18] Wang Y., Ma S., and Ruzzo W. L.. Spatial modeling of prostate cancer metabolic gene expression reveals extensive heterogeneity and selective vulnerabilities. Scientific Reports, 10:3490, Feb. 2020. ISSN 2045–2322. doi:10.1038/s41598-020-60384-w. URL https://www.ncbi.nlm.nih.gov/pmc/articles/PMC7044328/. 32103057 PMC7044328

[19] Navarro J. F., Croteau D. L., Jurek A., Andrusivova Z., Yang B., Wang Y., Ogedegbe B., Riaz T., Stoen M., Desler C., Rasmussen L. J., Tonjum T., Galas M.-C., Lundeberg J., and Bohr V. A.. Spatial Transcriptomics Reveals Genes Associated with Dysregulated Mitochondrial Functions and Stress Signaling in Alzheimer Disease. iScience, 23(10), Oct. 2020. ISSN 2589–0042. doi:10.1016/j.isci.2020.101556. URL https://www.cell.com/iscience/abstract/S2589-0042(20)30748-3. PMC7522123 33083725

[20] Saha A. and Datta A.. BRISC: bootstrap for rapid inference on spatial covariances: Rapid bootstrap for spatial covariances. Stat, 7(1):e184, 2018. ISSN 20491573. doi:10.1002/sta4.184. URL 10.1002/sta4.184.

[21] Eling N., Richard A. C., Richardson S., Marioni J. C., and Vallejos C. A.. Correcting the Mean-Variance Dependency for Differential Variability Testing Using Single-Cell RNA Sequencing Data. Cell Systems, 7(3):284–294.e12, Sept. 2018. ISSN 2405–4712. doi:10.1016/j.cels.2018.06.011. URL https://www.ncbi.nlm.nih.gov/pmc/articles/PMC6167088/. 30172840 PMC6167088

[22] Ahlmann-Eltze C. and Huber W.. Comparison of transformations for single-cell RNA-seq data. Nature Methods, 20(5):665–672, May 2023. ISSN 1548–7105. doi:10.1038/s41592-023-01814-1. URL https://www.nature.com/articles/s41592-023-01814-1. 37037999 PMC10172138

[23] Brennecke P., Anders S., Kim J. K., Kol odziejczyk A. A., Zhang X., Proserpio V., Baying B., Benes V., Teichmann S. A., Marioni J. C., and Heisler M. G.. Accounting for technical noise in single-cell RNA-seq experiments. Nature Methods, 10(11):1093–1095, Nov. 2013. ISSN 1548–7105. doi:10.1038/nmeth.2645. URL https://www.nature.com/articles/nmeth.2645. 24056876

[24] Antolović V., Miermont A., Corrigan A. M., and Chubb J. R.. Generation of Single-Cell Transcript Variability by Repression. Current Biology, 27(12):1811, June 2017. doi:10.1016/j.cub.2017.05.028. URL https://www.ncbi.nlm.nih.gov/pmc/articles/PMC5483230/. 28602650 PMC5483230

[25] Love M. I., Huber W., and Anders S.. Moderated estimation of fold change and dispersion for RNA-seq data with DESeq2. Genome Biology, 15(12):550, 2014. ISSN 1474–760X. doi:10.1186/s13059-014-0550-8. 25516281 PMC4302049

[26] Ritchie M. E., Phipson B., Wu D., Hu Y., Law C. W., Shi W., and Smyth G. K.. limma powers differential expression analyses for RNA-sequencing and microarray studies. Nucleic Acids Research, 43(7):e47, Apr. 2015. ISSN 1362–4962. doi:10.1093/nar/gkv007. 25605792 PMC4402510

[27] Robinson M. D., McCarthy D. J., and Smyth G. K.. edgeR : a Bioconductor package for differential expression analysis of digital gene expression data. Bioinformatics, 26(1):139–140, Jan. 2010. ISSN 1367–4811, 1367–4803. doi:10.1093/bioinformatics/btp616. URL https://academic.oup.com/bioinformatics/article/26/1/139/182458. 19910308 PMC2796818

[28] Townes F. W., Hicks S. C., Aryee M. J., and Irizarry R. A.. Feature selection and dimension reduction for single-cell RNA-Seq based on a multinomial model. Genome Biology, 20(1):295, Dec. 2019. ISSN 1474–760X. doi:10.1186/s13059-019-1861-6. URL 10.1186/s13059-019-1861-6. 31870412 PMC6927135

[29] Hao Y., Hao S., Andersen-Nissen E., Mauck W. M., Zheng S., Butler A., Lee M. J., Wilk A. J., Darby C., Zager M., Hoffman P., Stoeckius M., Papalexi E., Mimitou E. P., Jain J., Srivastava A., Stuart T., Fleming L. M., Yeung B., Rogers A. J., McElrath J. M., Blish C. A., Gottardo R., Smibert P., and Satija R.. Integrated analysis of multimodal single-cell data. Cell, 184(13):3573–3587.e29, June 2021. ISSN 00928674. doi:10.1016/j.cell.2021.04.048. URL https://linkinghub.elsevier.com/retrieve/pii/S0092867421005833. 34062119 PMC8238499

[30] Hafemeister C. and Satija R.. Normalization and variance stabilization of single-cell RNA-seq data using regularized negative binomial regression. Genome Biology, 20(1):296, Dec. 2019. ISSN 1474–760X. doi:10.1186/s13059-019-1874-1. URL 10.1186/s13059-019-1874-1. 31870423 PMC6927181

[31] Edsgärd D., Johnsson P., and Sandberg R.. Identification of spatial expression trends in single-cell gene expression data. Nature Methods, 15(5):339–342, May 2018. ISSN 1548–7105. doi:10.1038/nmeth.4634. 29553578 PMC6314435

[32] Booeshaghi A. S. and Pachter L.. Normalization of single-cell RNA-seq counts by $\log(x+1)$ or $\log(1+x)$. Bioinformatics, 37(15):2223–2224, Mar. 2021. ISSN 1367–4803. doi:10.1093/bioinformatics/btab085. URL https://www.ncbi.nlm.nih.gov/pmc/articles/PMC7989636/. 33676365 PMC7989636

[33] Law C. W., Chen Y., Shi W., and Smyth G. K.. voom: precision weights unlock linear model analysis tools for RNA-seq read counts. Genome Biology, 15(2):R29, 2014. ISSN 1465–6906. doi:10.1186/gb-2014-15-2-r29. URL 10.1186/gb-2014-15-2-r29. 24485249 PMC4053721

[34] Buettner F., Natarajan K. N., Casale F. P., Proserpio V., Scialdone A., Theis F. J., Teichmann S. A., Marioni J. C., and Stegle O.. Computational analysis of cell-to-cell heterogeneity in single-cell RNA-sequencing data reveals hidden subpopulations of cells. Nature Biotechnology, 33(2):155–160, Feb. 2015. ISSN 1546–1696. doi:10.1038/nbt.3102. 25599176

[35] Yang K., Tu J., and Chen T.. Homoscedasticity: an overlooked critical assumption for linear regression. General Psychiatry, 32(5):e100148, Oct. 2019. ISSN 2517–729X. doi:10.1136/gpsych-2019-100148. URL https://www.ncbi.nlm.nih.gov/pmc/articles/PMC6802968/. 31673679 PMC6802968

[36] Sun S., Zhu J., and Zhou X.. Statistical analysis of spatial expression patterns for spatially resolved transcriptomic studies. Nature Methods, 17(2):193–200, Feb. 2020. ISSN 1548–7105. doi:10.1038/s41592-019-0701-7. URL https://www.nature.com/articles/s41592-019-0701-7. 31988518 PMC7233129

[37] Datta A., Banerjee S., Finley A. O., and Gelfand A. E.. Hierarchical Nearest-Neighbor Gaussian Process Models for Large Geostatistical Datasets. Journal of the American Statistical Association, 111(514):800–812, Apr. 2016. ISSN 0162–1459, 1537–274X. doi:10.1080/01621459.2015.1044091. URL 10.1080/01621459.2015.1044091. 29720777 PMC5927603

[38] Finley A. O., Datta A., Cook B. C., Morton D. C., Andersen H. E., and Banerjee S.. Efficient algorithms for Bayesian Nearest Neighbor Gaussian Processes. Journal of Computational and Graphical Statistics: A Joint Publication of American Statistical Association, Institute of Mathematical Statistics, Interface Foundation of North America, 28(2):401–414, 2019. ISSN 1061–8600. doi:10.1080/10618600.2018.1537924. 31543693 PMC6753955

[39] 10x Genomics. Human Breast Cancer: Visium Fresh Frozen, Whole Transcriptome, July 2022. URL https://www.10xgenomics.com/resources/datasets/human-breast-cancer-visium-fresh-frozen-whole-transcriptome-1-standard.

[40] 10x Genomics. Human Breast Cancer: Whole Transcriptome Analysis, Oct. 2020. URL https://www.10xgenomics.com/datasets/human-breast-cancer-whole-transcriptome-analysis-1-standard-1-2-0.

[41] Wu S. Z., Al-Eryani G., Roden D., Junankar S., Harvey K., Andersson A., Thennavan A., Wang C., Torpy J., Bartonicek N., Wang T., Larsson L., Kaczorowski D., Weisenfeld N. I., Uytingco C. R., Chew J. G., Bent Z. W., Chan C.-L., Gnanasambandapillai V., Dutertre C.-A., Gluch L., Hui M. N., Beith J., Parker A., Robbins E., Segara D., Cooper C., Mak C., Chan B., Warrier S., Ginhoux F., Millar E., Powell J. E., Williams S. R., Liu X. S., O’Toole S., Lim E., Lundeberg J., Perou C. M., and Swarbrick A.. A single-cell and spatially resolved atlas of human breast cancers. Nature genetics, 53(9):1334–1347, Sept. 2021. ISSN 1061–4036. doi:10.1038/s41588-021-00911-1. URL https://www.ncbi.nlm.nih.gov/pmc/articles/PMC9044823/. 34493872 PMC9044823

[42] Weber L. M., Divecha H. R., Tran M. N., Kwon S. H., Spangler A., Montgomery K. D., Tippani M., Bharadwaj R., Kleinman J. E., Page S. C., Hyde T. M., Collado-Torres L., Maynard K. R., Martinowich K., and Hicks S. C.. The gene expression landscape of the human locus coeruleus revealed by single-nucleus and spatially-resolved transcriptomics. eLife, 12, Feb. 2023. doi:10.7554/eLife.84628. URL https://elifesciences.org/reviewed-preprints/84628. PMC10945708 38266073

[43] Denisenko E., de Kock L., Tan A., Beasley A. B., Beilin M., Jones M. E., Hou R., Muiri D. O., Bilic S., Mohan G. R. K. A., Salfinger S., Fox S., Hmon K. P. W., Yeow Y., Kim Y., John R., Gilderman T. S., Killingbeck E., Gray E. S., Cohen P. A., Yu Y., and Forrest A. R. R.. Spatial transcriptomics reveals discrete tumour microenvironments and autocrine loops within ovarian cancer subclones. Nature Communications, 15(1):2860, Apr. 2024. ISSN 2041–1723. doi:10.1038/s41467-024-47271-y. URL https://www.nature.com/articles/s41467-024-47271-y. PMC10991508 38570491

[44] Moran P. A. P.. NOTES ON CONTINUOUS STOCHASTIC PHENOMENA. Biometrika, 37(1–2):17–23, 1950. ISSN 0006–3444, 1464–3510. doi:10.1093/biomet/37.1-2.17. URL 10.1093/biomet/37.1-2.17. 15420245

[45] Chang Y., Liu J., Ma A., Li Z., Liu B., and Ma Q.. SpaGFT is a graph Fourier transform for tissue module identification from spatially resolved transcriptomics, Dec. 2022. URL 10.1101/2022.12.10.519929v1.

[46] Kats I., Vento-Tormo R., and Stegle O.. SpatialDE2: Fast and localized variance component analysis of spatial transcriptomics, Nov. 2021. URL 10.1101/2021.10.27.466045v2.

[47] Tsagris M. and Papadakis M.. Taking R to its limits: 70+ tips, Mar. 2018. URL https://peerj.com/preprints/26605v1.

[48] Chen C., Kim H. J., and Yang P.. Evaluating spatially variable gene detection methods for spatial transcriptomics data. Genome Biology, 25(1):18, Jan. 2024. ISSN 1474–760X. doi:10.1186/s13059-023-03145-y. URL 10.1186/s13059-023-03145-y. 38225676 PMC10789051

[49] Chen X., Ran Q., Tang J., Chen Z., Huang S., Shi X., and Xi R.. Benchmarking algorithms for spatially variable gene identification in spatial transcriptomics, July 2024. URL 10.1101/2024.07.04.602147. PMC12036962 40139667

[50] Opławski M., Srednicka A., Niewiadomska E., Boron D., Januszyk P., and Grabarek B. O.. Clinical and molecular evaluation of patients with ovarian cancer in the context of drug resistance to chemotherapy. Frontiers in Oncology, 12:954008, Aug. 2022. ISSN 2234–943X. doi:10.3389/fonc.2022.954008. URL https://www.ncbi.nlm.nih.gov/pmc/articles/PMC9389532/. 35992817 PMC9389532

[51] Xu Z., Li X., Li H., Nie C., Liu W., Li S., Liu Z., Wang W., and Wang J.. Suppression of DDX39B sensitizes ovarian cancer cells to DNA-damaging chemotherapeutic agents via destabilizing BRCA1 mRNA. Oncogene, 39(47):7051–7062, Nov. 2020. ISSN 1476–5594. doi:10.1038/s41388-020-01482-x. URL https://www.nature.com/articles/s41388-020-01482-x. 32989256

[52] Abrar M. A., Kaykobad M., Rahman M. S., and Samee M. A. H.. NoVaTeST: identifying genes with location-dependent noise variance in spatial transcriptomics data. Bioinformatics, 39(6):btad372, June 2023. ISSN 1367–4811. doi:10.1093/bioinformatics/btad372. URL 10.1093/bioinformatics/btad372. 37285319 PMC10283152

[53] Yan G., Hua S. H., and Li J. J.. Categorization of 33 computational methods to detect spatially variable genes from spatially resolved transcriptomics data, 2024. URL https://arxiv.org/abs/2405.18779. 10.1038/s41467-025-56080-w PMC11779979 39880807

[54] Zappia L., Phipson B., and Oshlack A.. Splatter: simulation of single-cell RNA sequencing data. Genome Biology, 18(1):174, Dec. 2017. ISSN 1474–760X. doi:10.1186/s13059-017-1305-0. URL 10.1186/s13059-017-1305-0. 28899397 PMC5596896

[55] Milne R. L., Burwinkel B., Michailidou K., Arias-Perez J.-I., Zamora M. P., Menéndez-Rodríguez P., Hardisson D., Mendiola M., González-Neira A., Pita G., Alonso M. R., Dennis J., Wang Q., Bolla M. K., Swerdlow A., Ashworth A., Orr N., Schoemaker M., Ko Y.-D., Brauch H., Hamann U., Andrulis I. L., Knight J. A., Glendon G., Tchatchou S., Matsuo K., Ito H., Iwata H., Tajima K., Li J., Brand J. S., Brenner H., Dieffenbach A. K., Arndt V., Stegmaier C., Lambrechts D., Peuteman G., Christiaens M.-R., Smeets A., Jakubowska A., Lubinski J., Jaworska-Bieniek K., Durda K., Hartman M., Hui M., Yen Lim W., Wan Chan C., Marme F., Yang R., Bugert P., Lindblom A., Margolin S., García-Closas M., Chanock S. J., Lissowska J., Figueroa J. D., Bojesen S. E., Nordestgaard B. G., Flyger H., Hooning M. J., Kriege M., van den Ouweland A. M., Koppert L. B., Fletcher O., Johnson N., dos Santos-Silva I., Peto J., Zheng W., Deming-Halverson S., Shrubsole M. J., Long J., Chang-Claude J., Rudolph A., Seibold P., Flesch-Janys D., Winqvist R., Pylkäs K., Jukkola-Vuorinen A., Grip M., Cox A., Cross S. S., Reed M. W., Schmidt M. K., Broeks A., Cornelissen S., Braaf L., Kang D., Choi J.-Y., Park S. K., Noh D.-Y., Simard J., Dumont M., Goldberg M. S., Labrèche F., Fasching P. A., Hein A., Ekici A. B., Beckmann M. W., Radice P., Peterlongo P., Azzollini J., Barile M., Sawyer E., Tomlinson I., Kerin M., Miller N., Hopper J. L., Schmidt D. F., Makalic E., Southey M. C., Hwang Teo S., Har Yip C., Sivanandan K., Tay W.-T., Shen C.-Y., Hsiung C.-N., Yu J.-C., Hou M.-F., Guénel P., Truong T., Sanchez M., Mulot C., Blot W., Cai Q., Nevanlinna H., Muranen T. A., Aittomäki K., Blomqvist C., Wu A. H., Tseng C.-C., Van Den Berg D., Stram D. O., Bogdanova N., Dörk T., Muir K., Lophatananon A., Stewart-Brown S., Siriwanarangsan P., Mannermaa A., Kataja V., Kosma V.-M., Hartikainen J. M., Shu X.-O., Lu W., Gao Y.-T., Zhang B., Couch F. J., Toland A. E., Yannoukakos D., Sangrajrang S., McKay J., Wang X., Olson J. E., Vachon C., Purrington K., Severi G., Baglietto L., Haiman C. A., Henderson B. E., Schumacher F., Le Marchand L., Devilee P., Tollenaar R. A., Seynaeve C., Czene K., Eriksson M., Humphreys K., Darabi H., Ahmed S., Shah M., Pharoah P. D., Hall P., Giles G. G., Benítez J., Dunning A. M., Chenevix-Trench G., Easton D. F., Berchuck A., Eeles R. A., Olama A. A. A., Kote-Jarai Z., Benlloch S., Antoniou A., McGuffog L., Offit K., Lee A., Dicks E., Luccarini C., Tessier D. C., Bacot F., Vincent D., LaBoissière S., Robidoux F., Nielsen S. F., Cunningham J. M., Windebank S. A., Hilker C. A., Meyer J., Angelakos M., Maskiell J., van der Schoot E., Rutgers E., Verhoef S., Hogervorst F., Boonyawongviroj P., Siriwanarungsan P., Schrauder M., Rübner M., Oeser S., Landrith S., Williams E., Ryder-Mills E., Sargus K., McInerney N., Colleran G., Rowan A., Jones A., Sohn C., Schneeweiß A., Bugert P., Álvarez N., Lacey J., Wang S., Ma H., Lu Y., Deapen D., Pinder R., Lee E., Schumacher F., Horn-Ross P., Reynolds P., Nelson D., Ziegler H., Wolf S., Hermann V., Lo W.-Y., Justenhoven C., Baisch C., Fischer H.-P., Brüning T., Pesch B., Rabstein S., Lotz A., Harth V., Heikkinen T., Erkkilä I., Aaltonen K., von Smitten K., Antonenkova N., Hillemanns P., Christiansen H., Myöhänen E., Kemiläinen H., Thorne H., Niedermayr E., Bowtell D., Chenevix-Trench G., deFazio A., Gertig D., Green A., Webb P., Green A., Parsons P., Hayward N., Webb P., Whiteman D., Fung A., Yashiki J., Peuteman G., Smeets D., Brussel T. V., Corthouts K., Obi N., Heinz J., Behrens S., Eilber U., Celik M., Olchers T., Manoukian S., Peissel B., Scuvera G., Zaffaroni D., Bonanni B., Feroce I., Maniscalco A., Rossi A., Bernard L., Tranchant M., Valois M.-F., Turgeon A., Heguy L., Sze Yee P., Kang P., Nee K. I., Mariapun S., Sook-Yee Y., Lee D., Ching T. Y., Taib N. A. M., Otsukka M., Mononen K., Selander T., Weerasooriya N., staff O., Krol-Warmerdam E., Molenaar J., Blom J., Brinton L., Szeszenia-Dabrowska N., Peplonska B., Zatonski W., Chao P., Stagner M., Bos P., Blom J., Crepin E., Nieuwlaat A., Heemskerk A., Higham S., Cross S., Cramp H., Connley D., Balasubramanian S., Brock I., Luccarini C., Conroy D., Baynes C., and Chua K.. Common non-synonymous SNPs associated with breast cancer susceptibility: findings from the Breast Cancer Association Consortium. Human Molecular Genetics, 23(22):6096–6111, Nov. 2014. ISSN 0964–6906. doi:10.1093/hmg/ddu311. URL https://www.ncbi.nlm.nih.gov/pmc/articles/PMC4204770/. 24943594 PMC4204770

[56] Jusino S., Rivera-Rivera Y., Chardón-Colón C., Ruiz-Justiz A. J., Vélez-Velázquez J., Isidro A., Cruz-Robles M. E., Bonilla-Claudio M., Armaiz-Pena G. N., and Saavedra H. I.. E2F3 drives the epithelial-to-mesenchymal transition, cell invasion, and metastasis in breast cancer. Experimental Biology and Medicine, 246(19):2057–2071, Oct. 2021. ISSN 1535–3702, 1535–3699. doi:10.1177/15353702211035693. URL 10.1177/15353702211035693. 34365840 PMC8524769

[57] Jin Y., Zhai M., Cao R., Yu H., Wu C., and Liu Y.. Silencing MFHAS1 Induces Pyroptosis via the JNK-activated NF-KB/Caspase1/ GSDMD Signal Axis in Breast Cancer. Current Pharmaceutical Design, 29(42):3408–3420, 2023. ISSN 1873–4286. doi:10.2174/0113816128268130231026054649. 37936452

[58] Yang W., Han B., Chen Y., and Geng F.. SAAL1, a novel oncogene, is associated with prognosis and immunotherapy in multiple types of cancer. Aging (Albany NY), 14(15):6316, Aug. 2022. doi:10.18632/aging.204224. URL https://www.ncbi.nlm.nih.gov/pmc/articles/PMC9417231/. 35963646 PMC9417231

[59] Jiao X., Hooper S. D., Djureinovic T., Larsson C., Wärnberg F., Tellgren-Roth C., Botling J., and Sjöblom T.. Gene rearrangements in hormone receptor negative breast cancers revealed by mate pair sequencing. BMC Genomics, 14:165, Mar. 2013. ISSN 1471–2164. doi:10.1186/1471-2164-14-165. URL https://www.ncbi.nlm.nih.gov/pmc/articles/PMC3600027/. 23496902 PMC3600027

[60] Li L., Li X., Qi L., Rychahou P., Jafari N., and Huang C.. The role of talin2 in breast cancer tumorigenesis and metastasis. Oncotarget, 8(63):106876, Dec. 2017. doi:10.18632/oncotarget.22449. URL https://www.ncbi.nlm.nih.gov/pmc/articles/PMC5739781/. 29290996 PMC5739781

[61] Yin Q., Wyatt C. J., Han T., Smalley K. S., and Wan L.. ITCH as a potential therapeutic target in human cancers. Seminars in cancer biology, 67(Pt 2):117–130, Dec. 2020. ISSN 1044–579X. doi:10.1016/j.semcancer.2020.03.003. URL https://www.ncbi.nlm.nih.gov/pmc/articles/PMC7724637/. 32165318 PMC7724637

[62] You Y., Ma Y., Wang Q., Ye Z., Deng Y., and Bai F.. Attenuated ZHX3 expression serves as a potential biomarker that predicts poor clinical outcomes in breast cancer patients. Cancer Management and Research, 11:1199–1210, Feb. 2019. ISSN 1179–1322. doi:10.2147/CMAR.S184340. URL https://www.ncbi.nlm.nih.gov/pmc/articles/PMC6368119/. 30787639 PMC6368119

[63] Wei Y., Zhang D., Shi H., Qian H., Chen H., Zeng Q., Jin F., Ye Y., Ou Z., Guo M., Guo B., and Chen T.. PDK1 promotes breast cancer progression by enhancing the stability and transcriptional activity of HIF-1alpha. Genes & Diseases, 11(4):101041, July 2024. ISSN 2352–3042. doi:10.1016/j.gendis.2023.06.013. URL https://www.sciencedirect.com/science/article/pii/S2352304223003112. 38560503 PMC10978537

[64] Zhu D., Zhao Z., Cui G., Chang S., Hu L., See Y. X., Lim M. G. L., Guo D., Chen X., Poudel B., Robson P., Luo Y., and Cheung E.. Single-Cell Transcriptome Analysis Reveals Estrogen Signaling Coordinately Augments One-Carbon, Polyamine, and Purine Synthesis in Breast Cancer. Cell Reports, 25(8):2285–2298.e4, Nov. 2018. ISSN 22111247. doi:10.1016/j.celrep.2018.10.093. URL https://linkinghub.elsevier.com/retrieve/pii/S2211124718317145. 30463022

[65] Ma S., Ren N., and Huang Q.. rs10514231 Leads to Breast Cancer Predisposition by Altering ATP6AP1L Gene Expression. Cancers, 13(15):3752, July 2021. ISSN 2072–6694. doi:10.3390/cancers13153752. URL https://www.ncbi.nlm.nih.gov/pmc/articles/PMC8345087/. 34359652 PMC8345087

[66] Shinde A., Chandak N., Singh J., Roy M., Mane M., Tang X., Vasiyani H., Currim F., Gohel D., Shukla S., Goyani S., Saranga M. V., Brindley D. N., and Singh R.. TNF-alpha induced NF-KB mediated LYRM7 expression modulates the tumor growth and metastatic ability in breast cancer. Free Radical Biology and Medicine, 211:158–170, Feb. 2024. ISSN 0891–5849. doi:10.1016/j.freeradbiomed.2023.12.018. URL https://www.sciencedirect.com/science/article/pii/S0891584923011747. 38104742

[67] Wu H., Guo X., Jiao Y., Wu Z., and Lv Q.. TRIM35 ubiquitination regulates the expression of PKM2 tetramer and dimer and affects the malignant behaviour of breast cancer by regulating the Warburg effect. International Journal of Oncology, 61(6):144, Oct. 2022. ISSN 1019–6439. doi:10.3892/ijo.2022.5434. URL https://www.ncbi.nlm.nih.gov/pmc/articles/PMC9581112/. 36196894 PMC9581112

[68] Zhao T.-T., Jin F., Li J.-G., Xu Y.-Y., Dong H.-T., Liu Q., Xing P., Zhu G.-L., Xu H., Yin S.-C., and Miao Z.-F.. TRIM32 promotes proliferation and confers chemoresistance to breast cancer cells through activation of the NF-KB pathway. Journal of Cancer, 9(8):1349–1356, Apr. 2018. ISSN 1837–9664. doi:10.7150/jca.22390. URL https://www.ncbi.nlm.nih.gov/pmc/articles/PMC5929078/. 29721043 PMC5929078

[69] Ko Y.-H., Domingo-Vidal M., Roche M., Lin Z., Whitaker-Menezes D., Seifert E., Capparelli C., Tuluc M., Birbe R. C., Tassone P., Curry J. M., Navarro-Sabate A., Manzano A., Bartrons R., Caro J., and Martinez-Outschoorn U.. TP53-inducible Glycolysis and Apoptosis Regulator (TIGAR) Metabolically Reprograms Carcinoma and Stromal Cells in Breast Cancer. The Journal of Biological Chemistry, 291(51):26291–26303, Dec. 2016. ISSN 0021–9258. doi:10.1074/jbc.M116.740209. URL https://www.ncbi.nlm.nih.gov/pmc/articles/PMC5159492/. 27803158 PMC5159492

[70] Raghavan V. and Manasa D. B.. Identification and Analysis of Disease Target Network of Human MicroRNA and Predicting Promising Leads for ZNF439, a Potential Target for Breast Cancer. International Journal of Bioscience, Biochemistry and Bioinformatics, pages 358–362, 2012. ISSN 20103638. doi:10.7763/IJBBB.2012.V2.132. URL http://www.ijbbb.org/show-33-382-1.html.

[71] Salem Y., Yacov N., Propheta-Meiran O., Breitbart E., and Mendel I.. Newly characterized motile sperm domain-containing protein 2 promotes human breast cancer metastasis. International Journal of Cancer, 144(1):125–135, Jan. 2019. ISSN 1097–0215. doi:10.1002/ijc.31665. 29978511 PMC6588022

[72] Suh E. J., Kabir M. H., Kang U.-B., Lee J. W., Yu J., Noh D.-Y., and Lee C.. Comparative profiling of plasma proteome from breast cancer patients reveals thrombospondin-1 and BRWD3 as serological biomarkers. Experimental & Molecular Medicine, 44(1):36–44, Jan. 2012. ISSN 1226–3613. doi:10.3858/emm.2012.44.1.003. URL https://www.ncbi.nlm.nih.gov/pmc/articles/PMC3277896/. 22024541 PMC3277896

[73] He L., Yang J., Hao Y., Yang X., Shi X., Zhang D., Zhao D., Yan W., Bie X., Chen L., Chen G., Zhao S., Liu X., Zheng H., and Zhang K.. DDX20: A Multifunctional Complex Protein. Molecules, 28(20):7198, Oct. 2023. ISSN 1420–3049. doi:10.3390/molecules28207198. URL https://www.ncbi.nlm.nih.gov/pmc/articles/PMC10608988/. 37894677 PMC10608988

[74] Park U.-H., Kang M.-R., Kim E.-J., Kwon Y.-S., Hur W., Yoon S. K., Song B.-J., Park J. H., Hwang J.-T., Jeong J.-C., and Um S.-J.. ASXL2 promotes proliferation of breast cancer cells by linking ERalpha to histone methylation. Oncogene, 35(28):3742–3752, July 2016. ISSN 1476–5594. doi:10.1038/onc.2015.443. URL https://www.nature.com/articles/onc2015443. 26640146

[75] Li D., Li Y., Wu X., Li Q., Yu J., Gen J., and Zhang X.-L.. Knockdown of Mgat5 Inhibits Breast Cancer Cell Growth with Activation of CD4+ T Cells and Macrophages. The Journal of Immunology, 180(5):3158–3165, Mar. 2008. ISSN 0022–1767, 1550–6606. doi:10.4049/jimmunol.180.5.3158. URL https://journals.aai.org/jimmunol/article/180/5/3158/78739/Knockdown-of-Mgat5-Inhibits-Breast-Cancer-Cell. 18292539

[76] Wang P., Zhang Q., Zhang H., Shao J., Zhang H., and Wang Z.. Molecular and clinical characterization of ICOS expression in breast cancer through large-scale transcriptome data. PLOS ONE, 18(12):e0293469, Dec. 2023. ISSN 1932–6203. doi:10.1371/journal.pone.0293469. URL 10.1371/journal.pone.0293469. 38127899 PMC10734928

[77] Perez-Añorve I. X., Gonzalez-De la Rosa C. H., Soto-Reyes E., Beltran-Anaya F. O., Del Moral-Hernandez O., Salgado-Albarran M., Angeles-Zaragoza O., Gonzalez-Barrios J. A., Landero-Huerta D. A., Chavez-Saldaña M., Garcia-Carranca A., Villegas-Sepulveda N., and Arechaga-Ocampo E.. New insights into radioresistance in breast cancer identify a dual function of miR-122 as a tumor suppressor and oncomiR. Molecular Oncology, 13(5):1249–1267, May 2019. ISSN 1574–7891. doi:10.1002/1878-0261.12483. URL https://www.ncbi.nlm.nih.gov/pmc/articles/PMC6487688/. 30938061 PMC6487688

[78] Tian C., Zhou S., and Yi C.. High NUP43 expression might independently predict poor overall survival in luminal A and in HER2+ breast cancer. Future Oncology (London, England), 14(15):1431–1442, June 2018. ISSN 1744–8301. doi:10.2217/fon-2017-0690. 29402145

[79] Quereda V., Bayle S., Vena F., Frydman S. M., Monastyrskyi A., Roush W. R., and Duckett D. R.. Therapeutic Targeting of CDK12/CDK13 in Triple-Negative Breast Cancer. Cancer Cell, 36(5):545–558.e7, Nov. 2019. ISSN 15356108. doi:10.1016/j.ccell.2019.09.004. URL https://linkinghub.elsevier.com/retrieve/pii/S1535610819304246. 31668947

[80] Mohammed Zaidh S., Aher K. B., Bhavar G. B., Irfan N., Ahmed H. N., and Ismail Y.. Genes adaptability and NOL6 protein inhibition studies of fabricated flavan-3-ols lead skeleton intended to treat breast carcinoma. International Journal of Biological Macromolecules, 258:127661, Feb. 2024. ISSN 0141–8130. doi:10.1016/j.ijbiomac.2023.127661. URL https://www.sciencedirect.com/science/article/pii/S0141813023045592. 37898257

[81] Arko-Boham B., Owusu B. A., Aryee N. A., Blay R. M., Owusu E. D. A., Tagoe E. A., Adams A. R., Gyasi R. K., Adu-Aryee N. A., and Mahmood S.. Prospecting for Breast Cancer Blood Biomarkers: Death-Associated Protein Kinase 1 (DAPK1) as a Potential Candidate. Disease Markers, 2020:6848703, May 2020. ISSN 0278–0240. doi:10.1155/2020/6848703. URL https://www.ncbi.nlm.nih.gov/pmc/articles/PMC7267859/. 32566040 PMC7267859

[82] Huang N., Li P., Sun X., Tong L., Dong X., Zhang X., Duan J., Sheng X., and Xin H.. TRIM21 mediates the synergistic effect of Olaparib and Sorafenib by degrading BRCA1 through ubiquitination in TNBC. npj Breast Cancer, 9(1):1–11, Oct. 2023. ISSN 2374–4677. doi:10.1038/s41523-023-00588-1. URL https://www.nature.com/articles/s41523-023-00588-1. 37864041 PMC10589312

[83] Li W., Hu S., Han Z., and Jiang X.. YY1-Induced Transcriptional Activation of FAM111B Contributes to the Malignancy of Breast Cancer. Clinical Breast Cancer, 22(4):e417–e425, June 2022. ISSN 1526–8209. doi:10.1016/j.clbc.2021.10.008. URL https://www.sciencedirect.com/science/article/pii/S1526820921002986. 34802969

[84] Chen X., Peng H., Zhang Z., Yang C., Liu Y., Chen Y., Yu F., Wu S., and Cao L.. SPDYC serves as a prognostic biomarker related to lipid metabolism and the immune microenvironment in breast cancer. Immunologic Research, June 2024. ISSN 0257–277X, 1559–0755. doi:10.1007/s12026-024-09505-5. URL 10.1007/s12026-024-09505-5. 38890248

[85] Zhang P., Yang Y., Qian K., Li L., Zhang C., Fu X., Zhang X., Chen H., Liu Q., Cao S., and Cui J.. A novel tumor suppressor ZBTB1 regulates tamoxifen resistance and aerobic glycolysis through suppressing HER2 expression in breast cancer. Journal of Biological Chemistry, 295(41):14140–14152, Oct. 2020. ISSN 00219258. doi:10.1074/jbc.RA119.010759. URL https://linkinghub.elsevier.com/retrieve/pii/S0021925817498097. 32690611 PMC7549032

[86] Wang X., Zheng Y., and Wang Y.. PEAK1 promotes invasion and metastasis and confers drug resistance in breast cancer. Clinical and Experimental Medicine, 22(3):393–402, Aug. 2022. ISSN 1591–9528. doi:10.1007/s10238-021-00761-5. URL 10.1007/s10238-021-00761-5. 34554318 PMC9338157

[87] Peng Y., Liu X., Liu X., Cheng X., Xia L., Qin L., Guan S., Wang Y., Wu X., Wu J., Yan D., Liu J., Zhang Y., Sun L., Liang J., and Shang Y.. RCCD1 promotes breast carcinogenesis through regulating hypoxia-associated mitochondrial homeostasis. Oncogene, 42(50):3684–3697, Dec. 2023. ISSN 1476–5594. doi:10.1038/s41388-023-02877-2. URL https://www.nature.com/articles/s41388-023-02877-2. 37903896

[88] Niu Y., Lin Z., Wan A., Chen H., Liang H., Sun L., Wang Y., Li X., Xiong X.-f., Wei B., Wu X., and Wan G.. RNA N6-methyladenosine demethylase FTO promotes breast tumor progression through inhibiting BNIP3. Molecular Cancer, 18(1):46, Dec. 2019. ISSN 1476–4598. doi:10.1186/s12943-019-1004-4. URL 10.1186/s12943-019-1004-4. 30922314 PMC6437932

[89] Liang P., Zhang J., Wu Y., Zheng S., Xu Z., Yang S., Wang J., Ma S., Xiao L., Hu T., Jiang W., Huang C., Xing Q., Kundu M., and Wang B.. An ULK1/2-PXN mechanotransduction pathway suppresses breast cancer cell migration. EMBO reports, 24(11):e56850, Nov. 2023. ISSN 1469–221X, 1469–3178. doi:10.15252/embr.202356850. URL 10.15252/embr.202356850. 37846507 PMC10626438

[90] Lu Y., Xiao Y., Yang J., Su H., Zhang X., Su F., Tian B., Zhao D., Ling X., and Zhang T.. TRIM65 Promotes Malignant Cell Behaviors in Triple-Negative Breast Cancer by Impairing the Stability of LATS1 Protein. Oxidative Medicine and Cellular Longevity, 2022:1–16, Aug. 2022. ISSN 1942–0994, 1942–0900. doi:10.1155/2022/4374978. URL https://www.hindawi.com/journals/omcl/2022/4374978/. PMC9402307 36035221

[91] Man X., Li Q., Wang B., Zhang H., Zhang S., and Li Z.. DNMT3A and DNMT3B in Breast Tumorigenesis and Potential Therapy. Frontiers in Cell and Developmental Biology, 10:916725, May 2022. ISSN 2296–634X. doi:10.3389/fcell.2022.916725. URL 10.3389/fcell.2022.916725. 35620052 PMC9127442

[92] Camp N. J., Parry M., Knight S., Abo R., Elliott G., Rigas S. H., Balasubramanian S. P., Reed M. W. R., McBurney H., Latif A., Newman W. G., Cannon-Albright L. A., Evans D. G., and Cox A.. Fine-Mapping CASP8 Risk Variants in Breast Cancer. Cancer Epidemiology, Biomarkers & Prevention, 21(1):176–181, Jan. 2012. ISSN 1055–9965, 1538–7755. doi:10.1158/1055-9965.EPI-11-0845. URL https://aacrjournals.org/cebp/article/21/1/176/157359/Fine-Mapping-CASP8-Risk-Variants-in-Breast. PMC3253962 22056502

[93] Xu J., Su S. M., Zhang X., Chan U. I., Adhav R., Shu X., Liu J., Li J., Mo L., Wang Y., An T., Lei J. H., Miao K., Deng C.-X., and Xu X.. ATP11B inhibits breast cancer metastasis in a mouse model by suppressing externalization of nonapoptotic phosphatidylserine. Journal of Clinical Investigation, 132(5):e149473, Mar. 2022. ISSN 1558–8238. doi:10.1172/JCI149473. URL https://www.jci.org/articles/view/149473. 35025764 PMC8884903

[94] Kim S., Kim K., Ryu J., Ryu T., Lim J. H., Oh J., Min J., Jung C., Hamamoto R., Son M., Kim D., and Cho H.. The novel prognostic marker, EHMT2, is involved in cell proliferation via HSPD1 regulation in breast cancer. International Journal of Oncology, Oct. 2018. ISSN 1019–6439, 1791–2423. doi:10.3892/ijo.2018.4608. URL 10.3892/ijo.2018.4608. PMC6254934 30365075

[95] Pullikuth A. K., Routh E. D., Zimmerman K. D., Chifman J., Chou J. W., Soike M. H., Jin G., Su J., Song Q., Black M. A., Print C., Bedognetti D., Howard-McNatt M., O’Neill S. S., Thomas A., Langefeld C. D., Sigalov A. B., Lu Y., and Miller L. D.. Bulk and Single-Cell Profiling of Breast Tumors Identifies TREM-1 as a Dominant Immune Suppressive Marker Associated With Poor Outcomes. Frontiers in Oncology, 11:734959, Dec. 2021. ISSN 2234–943X. doi:10.3389/fonc.2021.734959. URL 10.3389/fonc.2021.734959. 34956864 PMC8692779

[96] Pei L., Li Y., Gu H., Wang S., Wu W., Fan S., Shi X., and Si X.. Identification of SMC2 and SMC4 as prognostic markers in breast cancer through bioinformatics analysis. Clinical and Translational Oncology, May 2024. ISSN 1699–3055. doi:10.1007/s12094-024-03521-5. URL 10.1007/s12094-024-03521-5. 38773061

[97] Viera M., Yip G. W. C., Shen H.-M., Baeg G. H., and Bay B. H.. Targeting CD82/KAI1 for Precision Therapeutics in Surmounting Metastatic Potential in Breast Cancer. Cancers, 13(17):4486, Sept. 2021. ISSN 2072–6694. doi:10.3390/cancers13174486. URL https://www.mdpi.com/2072-6694/13/17/4486. 34503296 PMC8431267

[98] Samanta D., Huang T. Y.-T., Shah R., Yang Y., Pan F., and Semenza G. L.. BIRC2 Expression Impairs Anti-Cancer Immunity and Immunotherapy Efficacy. Cell Reports, 32(8):108073, Aug. 2020. ISSN 22111247. doi:10.1016/j.celrep.2020.108073. URL https://linkinghub.elsevier.com/retrieve/pii/S2211124720310585. 32846130

[99] Hervouet E., Claude-Taupin A., Gauthier T., Perez V., Fraichard A., Adami P., Despouy G., Monnien F., Algros M.-P., Jouvenot M., Delage-Mourroux R., and Boyer-Guittaut M.. The autophagy GABARAPL1 gene is epigenetically regulated in breast cancer models. BMC Cancer, 15(1):729, Dec. 2015. ISSN 1471–2407. doi:10.1186/s12885-015-1761-4. URL 10.1186/s12885-015-1761-4. 26474850 PMC4609056

[100] Siouda M., Dujardin A. D., Barbollat-Boutrand L., Mendoza-Parra M. A., Gibert B., Ouzounova M., Bouaoud J., Tonon L., Robert M., Foy J.-P., Lavergne V., Manie S. N., Viari A., Puisieux A., Ichim G., Gronemeyer H., Saintigny P., and Mulligan P.. CDYL2 Epigenetically Regulates MIR124 to Control NF-KB/STAT3-Dependent Breast Cancer Cell Plasticity. *iScience*, 23(6):101141, June 2020. ISSN 25890042. doi:10.1016/j.isci.2020.101141. URL https://linkinghub.elsevier.com/retrieve/pii/S2589004220303266. PMC7251929 32450513

[101] Kang M.-H., Jeong K. J., Kim W. Y., Lee H. J., Gong G., Suh N., Győrffy B., Kim S., Jeong S.-Y., Mills G. B., and Park Y.-Y.. Musashi RNA-binding protein 2 regulates estrogen receptor 1 function in breast cancer. Oncogene, 36(12):1745–1752, Mar. 2017. ISSN 0950–9232, 1476–5594. doi:10.1038/onc.2016.327. URL https://www.nature.com/articles/onc2016327. 27593929

[102] Hu W.-F., Krieger K. L., Lagundžin D., Li X., Cheung R. S., Taniguchi T., Johnson K. R., Bessho T., Monteiro A. N. A., and Woods N. T.. CTDP1 regulates breast cancer survival and DNA repair through BRCT-specific interactions with FANCI. Cell Death Discovery, 5(1):105, June 2019. ISSN 2058–7716. doi:10.1038/s41420-019-0185-3. URL https://www.nature.com/articles/s41420-019-0185-3. 31240132 PMC6584691

[103] Zhang J., Li Y., Wang J.-G., Feng J.-Y., Huang G.-D., and Luo C.-G.. Dihydroartemisinin Affects STAT3/DDA1 Signaling Pathway and Reverses Breast Cancer Resistance to Cisplatin. The American Journal of Chinese Medicine, 51(02):445–459, Jan. 2023. ISSN 0192–415X, 1793–6853. doi:10.1142/S0192415X23500234. URL 10.1142/S0192415X23500234. 36891981

[104] Peng Y., Li H., Fu Y., Guo S., Qu C., Zhang Y., Zong B., and Liu S.. JAM2 predicts a good prognosis and inhibits invasion and migration by suppressing EMT pathway in breast cancer. International Immunopharmacology, 103:108430, Feb. 2022. ISSN 15675769. doi:10.1016/j.intimp.2021.108430. URL https://linkinghub.elsevier.com/retrieve/pii/S1567576921010663. 34923424

[105] Zhang S., Liu X., Chen W., Zhang K., Wu Q., and Wei Y.. Targeting TAF1 with BAY-299 induces antitumor immunity in triple-negative breast cancer. Biochemical and Biophysical Research Communications, 665:55–63, July 2023. ISSN 0006291X. doi:10.1016/j.bbrc.2023.04.100. URL https://linkinghub.elsevier.com/retrieve/pii/S0006291X23005314. 37148745

[106] Johnstone C. N., Pattison A. D., Harrison P. F., Powell D. R., Lock P., Ernst M., Anderson R. L., and Beilharz T. H.. FGF13 promotes metastasis of triple-negative breast cancer. International Journal of Cancer, 147(1):230–243, July 2020. ISSN 0020–7136, 1097–0215. doi:10.1002/ijc.32874. URL 10.1002/ijc.32874. 31957002

[107] Chen Z., Cui N., Zhao J.-s, Wu J.-f., Ma F, Li C., and Liu X.-y.. Expressions of ZNF436, beta-catenin, EGFR, and CMTM5 in breast cancer and their clinical significances. European Journal of Histochemistry, 65(1), Jan. 2021. ISSN 2038–8306, 1121–760X. doi:10.4081/ejh.2021.3173. URL https://www.ejh.it/index.php/ejh/article/view/3173. PMC7856825 33478201

[108] Ngubo M., Moradi F., Ito C. Y., and Stanford W. L.. Tissue-Specific Tumour Suppressor and Oncogenic Activities of the Polycomb-like Protein MTF2. Genes, 14(10):1879, Sept. 2023. ISSN 2073–4425. doi:10.3390/genes14101879. URL https://www.mdpi.com/2073-4425/14/10/1879. 37895228 PMC10606531

[109] Jin L., Luo C., Wu X., Li M., Wu S., and Feng Y.. LncRNA-HAGLR motivates triple negative breast cancer progression by regulation of WNT2 via sponging miR-335–3p. Aging, 13(15):19306–19316, Aug. 2021. ISSN 1945–4589. doi:10.18632/aging.203272. URL 10.18632/aging.203272. 34375306 PMC8386551

[110] Han H.-J., Russo J., Kohwi Y., and Kohwi-Shigematsu T.. SATB1 reprogrammes gene expression to promote breast tumour growth and metastasis. Nature, 452(7184):187–193, Mar. 2008. ISSN 0028–0836, 1476–4687. doi:10.1038/nature06781. URL https://www.nature.com/articles/nature06781. 18337816

[111] Erin N., Podnos A., Tanriover G., Duymus O., Cote E., Khatri I., and Gorczynski R. M.. Bidirectional effect of CD200 on breast cancer development and metastasis, with ultimate outcome determined by tumor aggressiveness and a cancer-induced inflammatory response. Oncogene, 34(29):3860–3870, July 2015. ISSN 0950–9232, 1476–5594. doi:10.1038/onc.2014.317. URL https://www.nature.com/articles/onc2014317. 25263452

[112] Tan P., Ye Y., He L., Xie J., Jing J., Ma G., Pan H., Han L., Han W., and Zhou Y.. TRIM59 promotes breast cancer motility by suppressing p62-selective autophagic degradation of PDCD10. PLOS Biology, 16(11):e3000051, Nov. 2018. ISSN 1545–7885. doi:10.1371/journal.pbio.3000051. URL 10.1371/journal.pbio.3000051. 30408026 PMC6245796

[113] Mondal M., Conole D., Nautiyal J., and Tate E. W.. UCHL1 as a novel target in breast cancer: emerging insights from cell and chemical biology. British Journal of Cancer, 126(1):24–33, Jan. 2022. ISSN 0007–0920, 1532–1827. doi:10.1038/s41416-021-01516-5. URL https://www.nature.com/articles/s41416-021-01516-5. 34497382 PMC8727673

[114] Chen D., Sun Y., Wei Y., Zhang P., Rezaeian A. H., Teruya-Feldstein J., Gupta S., Liang H., Lin H.-K., Hung M.-C., and Ma L.. LIFR is a breast cancer metastasis suppressor upstream of the Hippo-YAP pathway and a prognostic marker. Nature Medicine, 18(10):1511–1517, Oct. 2012. ISSN 1078–8956, 1546–170X. doi:10.1038/nm.2940. URL https://www.nature.com/articles/nm.2940. PMC3684419 23001183

[115] Ivanova I. A., Vermeulen J. F., Ercan C., Houthuijzen J. M., Saig F. A., Vlug E. J., Van Der Wall E., Van Diest P. J., Vooijs M., and Derksen P. W. B.. FER kinase promotes breast cancer metastasis by regulating alpha6- and beta1-integrin-dependent cell adhesion and anoikis resistance. Oncogene, 32(50):5582–5592, Dec. 2013. ISSN 0950–9232, 1476–5594. doi:10.1038/onc.2013.277. URL https://www.nature.com/articles/onc2013277. 23873028 PMC3898493

[116] Yang W., Li J., Zhang M., Yu H., Zhuang Y., Zhao L., Ren L., Gong J., Bi H., Zeng L., Xue Y., Yang J., Zhao Y., Wang S., Gao S., Fu Z., Li D., Zhang J., Wang T., Shan M., Tang B., and Li X.. Elevated expression of the rhythm gene NFIL3 promotes the progression of TNBC by activating NF-KB signaling through suppression of NFKBIA transcription. Journal of Experimental & Clinical Cancer Research, 41(1):67, Dec. 2022. ISSN 1756–9966. doi:10.1186/s13046-022-02260-1. URL 10.1186/s13046-022-02260-1. 35180863 PMC8855542

[117] Shropshire D. B., Acosta F. M., Fang K., Benavides J., Sun L.-Z., Jin V. X., and Jiang J. X.. Association of adenosine signaling gene signature with estrogen receptor-positive breast and prostate cancer bone metastasis. Frontiers in Medicine, 9:965429, Sept. 2022. ISSN 2296–858X. doi:10.3389/fmed.2022.965429. URL 10.3389/fmed.2022.965429. 36186774 PMC9520286

[118] Marrufo A. M., Mathew S. O., Chaudhary P., Malaer J. D., Vishwanatha J. K., and Mathew P. A.. Blocking LLT1 (CLEC2D, OCIL)-NKRP1A (CD161) interaction enhances natural killer cell-mediated lysis of triple-negative breast cancer cells. American Journal of Cancer Research, 8 (6):1050–1063, June 2018. ISSN 2156–6976. URL https://www.ncbi.nlm.nih.gov/pmc/articles/PMC6048397/. 30034942 PMC6048397

[119] Tai Y., Chow A., Han S., Coker C., Ma W., Gu Y., Estrada Navarro V., Kandpal M., Hibshoosh H., Kalinsky K., Manova-Todorova K., Safonov A., Walsh E. M., Robson M., Norton L., Baer R., Merghoub T., Biswas A. K., and Acharyya S.. FLT1 activation in cancer cells promotes PARP-inhibitor resistance in breast cancer. EMBO Molecular Medicine, 16(8):1957–1980, July 2024. ISSN 1757–4684. doi:10.1038/s44321-024-00094-2. URL 10.1038/s44321-024-00094-2. 38956205 PMC11319505

[120] Lee Y.-J., Ho S.-R., Graves J. D., Xiao Y., Huang S., and Lin W.-C.. CGRRF1, a growth suppressor, regulates EGFR ubiquitination in breast cancer. Breast Cancer Research, 21(1):134, Dec. 2019. ISSN 1465–542X. doi:10.1186/s13058-019-1212-2. URL 10.1186/s13058-019-1212-2. 31801577 PMC6894136

[121] Ekyalongo R. C., Mukohara T., Funakoshi Y., Tomioka H., Kataoka Y., Shimono Y., Chayahara N., Toyoda M., Kiyota N., and Minami H.. TYRO3 as a potential therapeutic target in breast cancer. Anticancer Research, 34(7):3337–3345, July 2014. ISSN 1791–7530. 24982338

[122] Cheng Y., Lin L., Li X., Lu A., Hou C., Wu Q., Hu X., Zhou Z., Chen Z., and Tang F.. ADAM10 is involved in the oncogenic process and chemo-resistance of triple-negative breast cancer via regulating Notch1 signaling pathway, CD44 and PrPc. Cancer Cell International, 21(1):32, Jan. 2021. ISSN 1475–2867. doi:10.1186/s12935-020-01727-5. URL 10.1186/s12935-020-01727-5. 33413403 PMC7791678

[123] Wei Y., Huang H., Qiu Z., Li H., Tan J., Ren G., and Wang X.. NLRP1 Overexpression Is Correlated with the Tumorigenesis and Proliferation of Human Breast Tumor. BioMed Research International, 2017:1–9, 2017. ISSN 2314–6133, 2314–6141. doi:10.1155/2017/4938473. URL https://www.hindawi.com/journals/bmri/2017/4938473/. PMC5682047 29214170

[124] Huang Z., Yang J., Qiu W., Huang J., Chen Z., Han Y., and Ye C.. HAUS5 Is A Potential Prognostic Biomarker With Functional Significance in Breast Cancer. Frontiers in Oncology, 12:829777, Feb. 2022. ISSN 2234–943X. doi:10.3389/fonc.2022.829777. URL 10.3389/fonc.2022.829777. 35280773 PMC8913513

[125] Tong D., Zhang J., Wang X., Li Q., Liu L. Y., Yang J., Guo B., Ni L., Zhao L., and Huang C.. MeCP2 facilitates breast cancer growth via promoting ubiquitination-mediated P53 degradation by inhibiting RPL5/RPL11 transcription. Oncogenesis, 9(5):56, June 2020. ISSN 2157–9024. doi:10.1038/s41389-020-0239-7. URL https://www.nature.com/articles/s41389-020-0239-7. 32483207 PMC7264296

[126] Spears M., Rabiasz G. J., Scott D., Ntougkos E., Fegan S., Miller E. P., Smyth J. F., and Sellar G. C.. The function of tumor suppressor genes in ovarian cancer: the role of LSAMP. Cancer Res, 66 (8_Supplement)(587), Apr. 2006. ISSN 1538–7445.

[127] Shi Y., Gao S., Zheng Y., Yao M., and Ruan F.. LncRNA CASC15 Functions As An Unfavorable Predictor Of Ovarian Cancer Prognosis And Inhibits Tumor Progression Through Regulation Of miR-221/ARID1A Axis. OncoTargets and therapy, 12:8725–8736, Oct. 2019. ISSN 1178–6930. doi:10.2147/OTT.S219900. URL https://www.ncbi.nlm.nih.gov/pmc/articles/PMC6815787/. 31695430 PMC6815787

[128] Liu A.-r., Liu Y.-n., Shen S.-x, Yan L.-r, Lv Z, Ding H.-x., Wang A., Yuan Y, and Xu Q. Comprehensive Analysis and Validation of Solute Carrier Family 25 (SLC25) and Its Correlation with Immune Infiltration in Pan-Cancer. BioMed Research International, 2022:1–23, Oct. 2022. ISSN 2314–6141, 2314–6133. doi:10.1155/2022/4009354. URL https://www.hindawi.com/journals/bmri/2022/4009354/. PMC9569204 36254139

[129] Wang J., Dean D. C., Hornicek F. J., Shi H., and Duan Z.. Cyclin-dependent kinase 9 (CDK9) is a novel prognostic marker and therapeutic target in ovarian cancer. The FASEB Journal, 33 (5):5990–6000, May 2019. ISSN 0892–6638, 1530–6860. doi:10.1096/fj.201801789RR. URL 10.1096/fj.201801789RR. 30726104 PMC6463912

[130] Gruosso T., Garnier C., Abelanet S., Kieffer Y., Lemesre V., Bellanger D., Bieche I., Marangoni E., Sastre-Garau X., Mieulet V., and Mechta-Grigoriou F.. MAP3K8/TPL-2/COT is a potential predictive marker for MEK inhibitor treatment in high-grade serous ovarian carcinomas. Nature Communications, 6(1):8583, Oct. 2015. ISSN 2041–1723. doi:10.1038/ncomms9583. URL https://www.nature.com/articles/ncomms9583. PMC4633961 26456302

[131] Wang S., Xia Y., Huang P., Xu C., Qian Y., Fang T., and Gao Q.. Suppression of GCH1 Sensitizes Ovarian Cancer and Breast Cancer to PARP Inhibitor. Journal of Oncology, 2023:1–16, Feb. 2023. ISSN 1687–8469, 1687–8450. doi:10.1155/2023/1453739. URL https://www.hindawi.com/journals/jo/2023/1453739/. PMC9925261 36793373

[132] Xie Y., Chen H., Shen P., Shen Q., and Luo Y.. PCYT2-Mediated Regulation of Phospholipid Metabolism Enhances Metastasis in Epithelial Ovarian Cancer via the AKT/mTOR and HIPPO Signaling Pathways. Journal of Biological Regulators and Homeostatic Agents, 38(2):1351–1364, Feb. 2024. ISSN 0393–974X. doi:10.23812/j.biol.regul.homeost.agents.20243802.108. URL 10.23812/j.biol.regul.homeost.agents.20243802.108.

