# Supplementary material for: Addressing the mean-variance relationship in spatially resolved transcriptomics data with spoon
Source: bioRxiv. 2024 Nov 8:2024.11.04.621867. Preprint. [Version 2] doi: 10.1101/2024.11.04.621867 (PMC11580860; doi:10.1101/2024.11.04.621867)
Supplement: Supplement 1 [file NIHPP2024.11.04.621867v2-supplement-1.pdf]

bioRxiv preprint doi: https://doi.org/10.1101/2024.11.04.621867; this version posted November 8, 2024. The copyright holder for this preprint (which was not certified by peer review) is the author/funder, who has granted bioRxiv a license to display the preprint in perpetuity. It is made available under aCC-BY 4.0 International license.

Supplementary Materials

Addressing the mean-variance relationship in spatially resolved transcriptomics data with *spoon*

Kinnary Shah, Boyi Guo, Stephanie C. Hicks*

*Correspondence to shicks19@jhu.edu

Contents

1. **Figures S1-S5**
2. **Tables S1-S8**

bioRxiv preprint doi: https://doi.org/10.1101/2024.11.04.621867; this version posted November 8, 2024. The copyright holder for this preprint (which was not certified by peer review) is the author/funder, who has granted bioRxiv a license to display the preprint in perpetuity. It is made available under aCC-BY 4.0 International license.

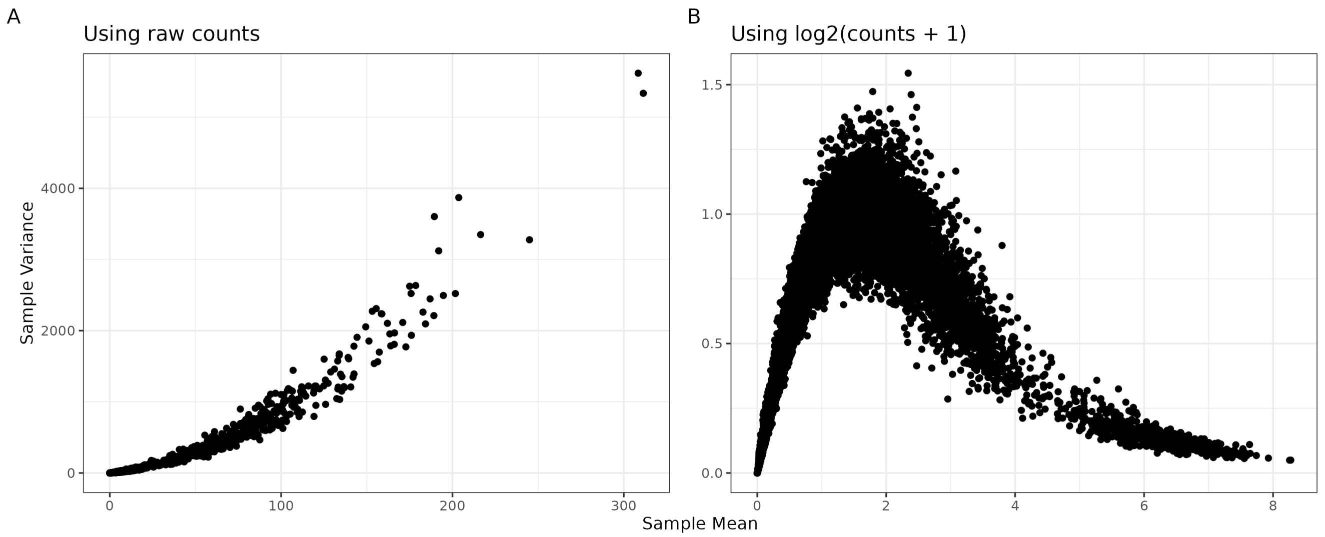

Figure S1: Visualizing the mean-variance relationship on different scales. The mean-variance relationship exists with or without a log-transformation. Gene expression counts were simulated using the `splatter` R/Bioconductor package [54] for G=10,000 genes and N=100 observations (or cells) under a Gamma-Poisson model. Each point represents one gene. Both representations illustrate the mean-variance relationship where the x-axis is the sample mean and y-axis is the sample variance using either (**A**) the raw counts or (**B**) the $\log_2$-transformed counts with a pseudocount of 1 (or $\log_2(\text{counts}+1)$). Here, the log-transformation overcorrects for the mean-variance relationship for the larger counts.

bioRxiv preprint doi: https://doi.org/10.1101/2024.11.04.621867; this version posted November 8, 2024. The copyright holder for this preprint (which was not certified by peer review) is the author/funder, who has granted bioRxiv a license to display the preprint in perpetuity. It is made available under aCC-BY 4.0 International license.

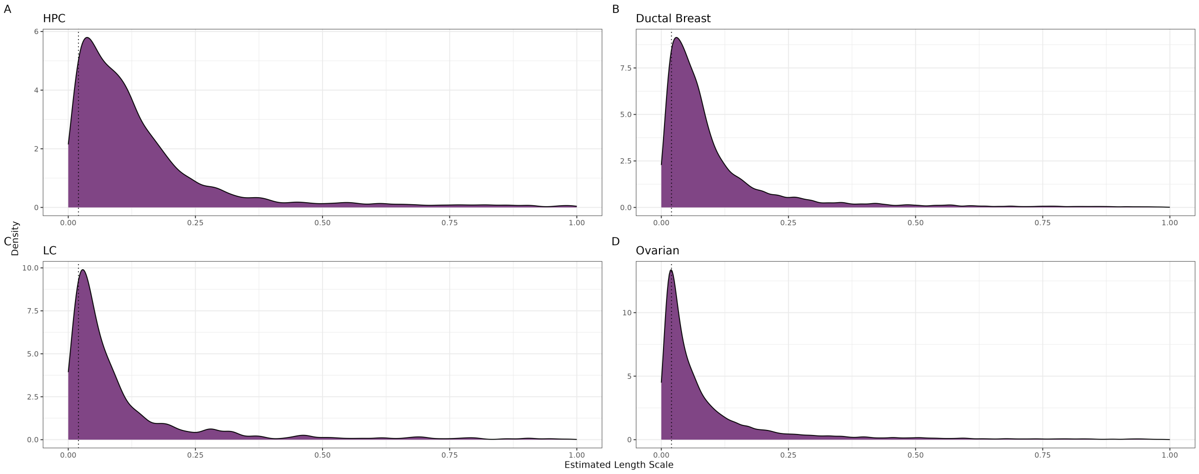

Figure S2: Real data estimated lengthscale distributions using `nnSVG`. This figure shows the estimated lengthscale distributions for four real datasets (**A**) HPC [16], (**B**) Ductal Breast cancer [39], (**C**) LC [42], and (**D**) Ovarian cancer [43]. For each dataset, `nnSVG` was used to calculate the estimated lengthscale value for each gene and the distribution of values between 0 and 1 is plotted. The dotted line highlights the lengthscale value used in the primary simulation evaluations.

bioRxiv preprint doi: https://doi.org/10.1101/2024.11.04.621867; this version posted November 8, 2024. The copyright holder for this preprint (which was not certified by peer review) is the author/funder, who has granted bioRxiv a license to display the preprint in perpetuity. It is made available under aCC-BY 4.0 International license.

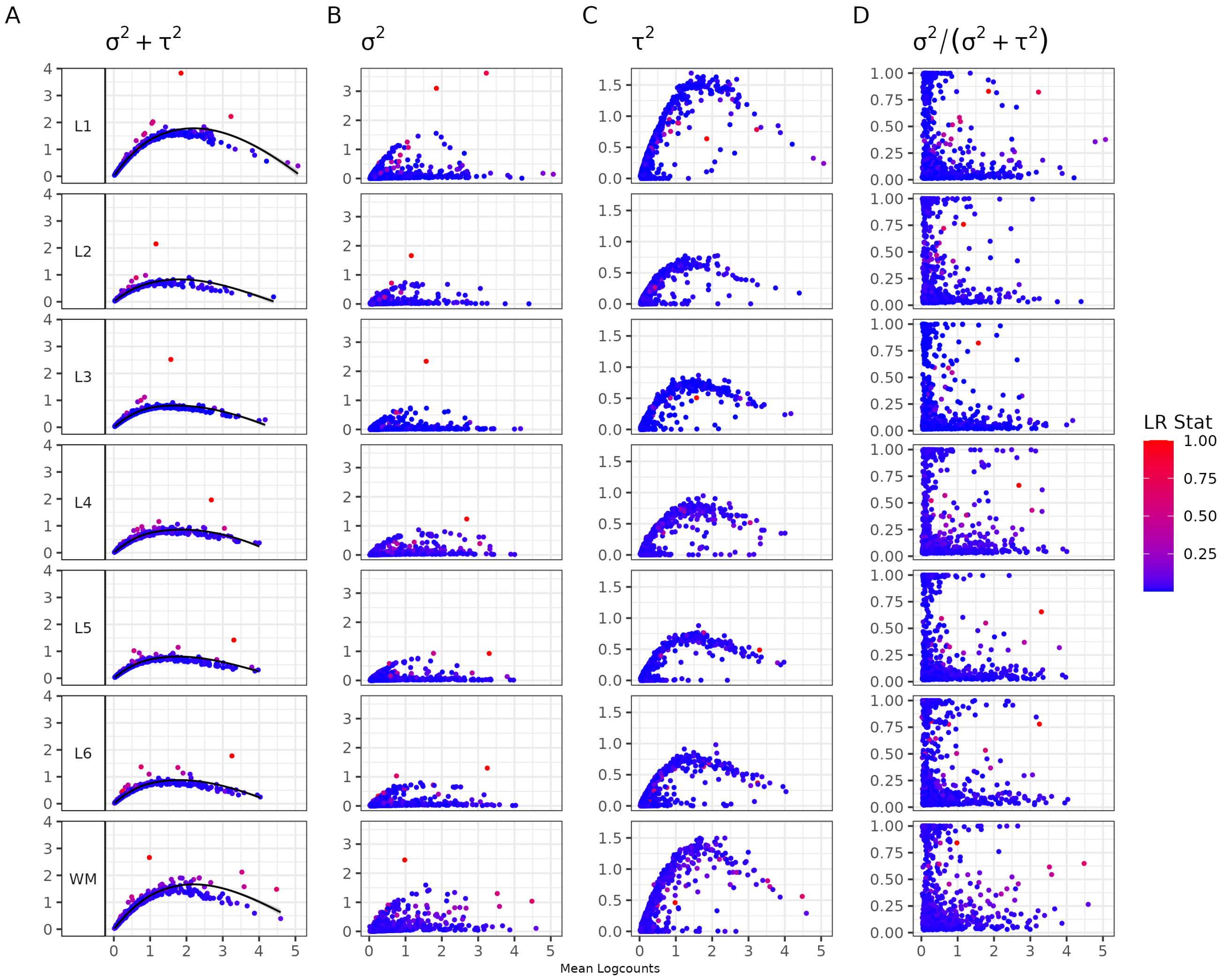

Figure S3: Mean-variance relationship after conditioning out biological variance measured by Gaussian process. Each row is a cortical layer from the DLPFC dataset, in order from top to bottom: Layers I-VI, white matter (WM). Each point is a gene colored by the likelihood ratio statistic (LR Stat) for a test comparing the fitted model against a classical linear model for the spatial component of variance. The likelihood ratio statistics are scaled by the maximum likelihood ratio statistic for each layer in order to have more uniform visualization. The x-axis represents mean logcounts and the y-axes represent different components of variance, in order from left to right: total variance $\sigma^2 + \tau^2$, spatial variance σ^2, nonspatial variance τ^2, and proportion of spatial variance $\sigma^2/(\sigma^2 + \tau^2)$.

bioRxiv preprint doi: https://doi.org/10.1101/2024.11.04.621867; this version posted November 8, 2024. The copyright holder for this preprint (which was not certified by peer review) is the author/funder, who has granted bioRxiv a license to display the preprint in perpetuity. It is made available under aCC-BY 4.0 International license.

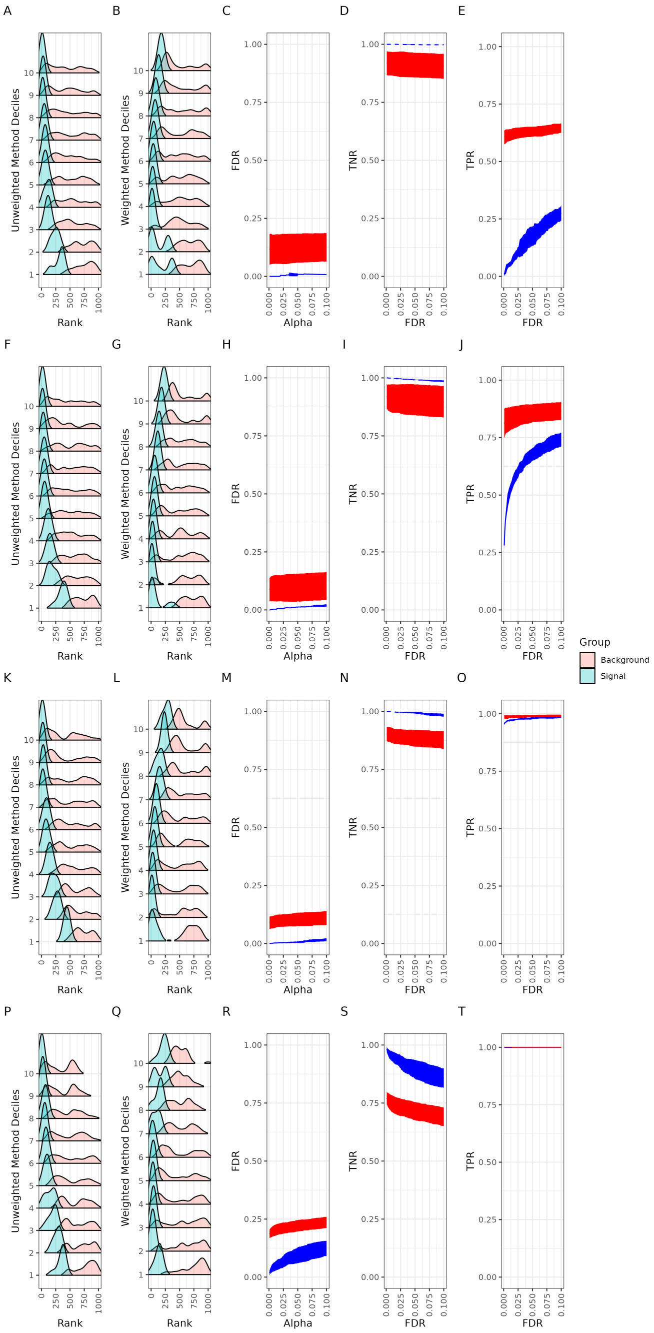

Figure S4: Removing the mean-variance relationship with expanded lengthscale metrics. This dataset contains 1,000 simulated genes across 968 spots. Each row represents a simulation setting with unique lengthscales, in order from top to bottom: 50, 60, 100, 500. Separately for unweighted and weighted methods, the genes were binned into deciles based on mean logcounts. Decile 1 is the lowest mean expression values. The first column of plots is unweighted ranks and the second column of plots is weighted ranks. Within each decile, the density of the top 10% ranks is plotted as the signal and the density of the remaining ranks is plotted as the background. The final three columns show the false discovery rate (FDR), true negative rate (TNR), and true positive rate (TPR). The red represents weighted nnSVG and the blue represents unweighted nnSVG. These plots represent the average of each respective rate over five iterations of the same simulation with unique random seeds.

bioRxiv preprint doi: https://doi.org/10.1101/2024.11.04.621867; this version posted November 8, 2024. The copyright holder for this preprint (which was not certified by peer review) is the author/funder, who has granted bioRxiv a license to display the preprint in perpetuity. It is made available under aCC-BY 4.0 International license.

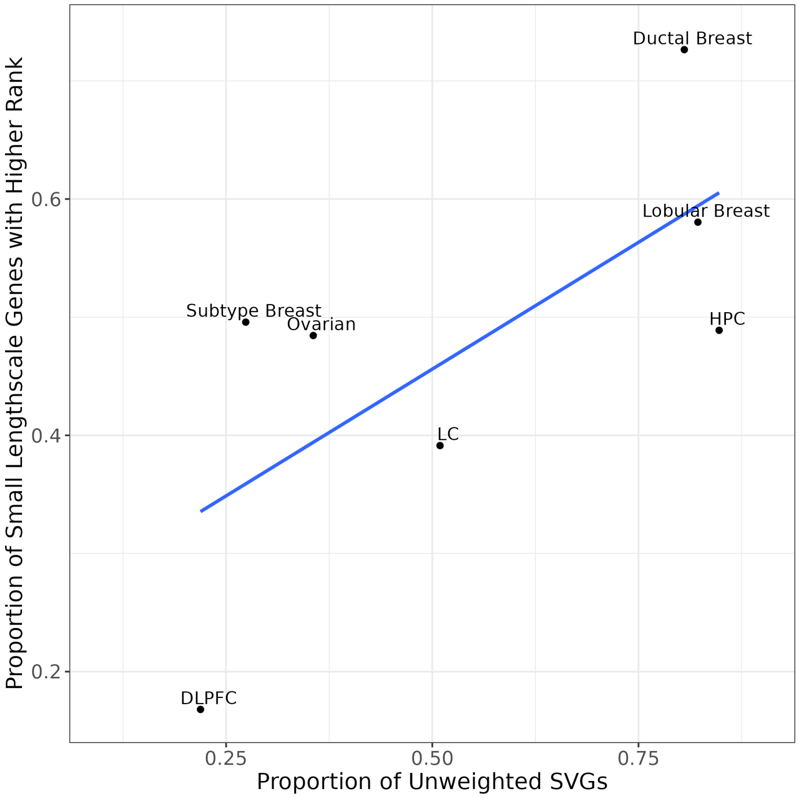

Figure S5: Ranking small lengthscale genes after weighting. Each point is a unique real dataset analyzed with 10x Genomics Visium. The x-axis is the proportion of SVGs from running unweighted `nnSVG` on the dataset. The y-axis is the proportion of genes with a small lengthscale (40-90) that are higher ranked in weighted `nnSVG` compared to unweighted `nnSVG`.

bioRxiv preprint doi: https://doi.org/10.1101/2024.11.04.621867; this version posted November 8, 2024. The copyright holder for this preprint (which was not certified by peer review) is the author/funder, who has granted bioRxiv a license to display the preprint in perpetuity. It is made available under aCC-BY 4.0 International license.

Table S1: Low Mean, Ductal Breast Genes Relation to Cancer. This table shows all genes with means less than the 25th percentile in the Ductal Breast [39] dataset which were in the lowest 10% of ranks before weighting and then increased to the highest 10% of ranks after weighting. The second column indicates if the gene is related to Breast cancer, with the corresponding reference in the third column.

Gene	Cancer-related?	References
TMEM39B		
ETAA1		
ATXN7	TRUE	[55]
BBS7		
MFSD8		
ETFDH		
E2F3	TRUE	[56]
FIG4		
TSPYL4		
METTL2B		
MFHAS1	TRUE	[57]
MAK16		
GKAP1		
SAAL1	TRUE	[58]
DDX10	TRUE	[59]
B3GLCT		
GNB5		
TLN2	TRUE	[60]
VPS33B		
ASB7		
MOSMO		
MED26		
ZNF227		
ITCH	TRUE	[61]
ZHX3	TRUE	[62]
ZBTB21		

Table S2: Low Mean, Subtype Breast Genes Relation to Cancer. This table shows all genes with means less than the 25th percentile in the Subtype Breast [41] dataset which were in the lowest 10% of ranks before weighting and then increased to the highest 10% of ranks after weighting. The second column indicates if the gene is related to Breast cancer, with the corresponding reference in the third column.

Gene	Cancer-related?	References
RELA-DT		

bioRxiv preprint doi: https://doi.org/10.1101/2024.11.04.621867; this version posted November 8, 2024. The copyright holder for this preprint (which was not certified by peer review) is the author/funder, who has granted bioRxiv a license to display the preprint in perpetuity. It is made available under aCC-BY 4.0 International license.

Table S3: Low Mean, Lobular Breast Genes Relation to Cancer. This table shows all genes with means less than the 25th percentile in the Lobular Breast [40] dataset which were in the lowest 10% of ranks before weighting and then increased to the highest 10% of ranks after weighting. The second column indicates if the gene is related to Breast cancer, with the corresponding reference in the third column.

Gene	Cancer-related?	References
TMEM51		
PDK1	TRUE	[63]
QTRT2		
OSBPL11		
TMEM44		
CPLX1		
PPAT	TRUE	[64]
ATP6AP1L	TRUE	[65]
LYRM7	TRUE	[66]
MSH5		
ZBTB24		
SHPRH		
TRIM35	TRUE	[67]
NCALD		
SNX30		
TRIM32	TRUE	[68]
FRAT1	TRUE	
RAB11FIP2		
TIGAR	TRUE	[69]
INTS13		
DLEU2		
ATXN1L		
SNHG30		
SEPTIN4		
ZNF439	TRUE	[70]
ZNF181		
TMEM191B		
ZNF74		
MOSPD2	TRUE	[71]
FAAH2		
BRWD3	TRUE	[72]

Table S4: Low Mean, Ovarian Genes Relation to Cancer. This table shows all genes with means less than the 25th percentile in the Ovarian [43] dataset which were in the lowest 10% of ranks before weighting and then increased to the highest 10% of ranks after weighting. The second column indicates if the gene is related to Ovarian cancer, with the corresponding reference in the third column.

Gene	Cancer-related?	References
TUFT1	TRUE	[50]
EHHADH		
DDX39B	TRUE	[51]
NAV2		
AC026471.4		
SMYD4		
HEXIM2		

bioRxiv preprint doi: https://doi.org/10.1101/2024.11.04.621867; this version posted November 8, 2024. The copyright holder for this preprint (which was not certified by peer review) is the author/funder, who has granted bioRxiv a license to display the preprint in perpetuity. It is made available under aCC-BY 4.0 International license.

Table S5: Small Lengthscale, Ductal Breast Genes Relation to Cancer. This table shows all genes with lengthscale values between 40 to 90 in the Ductal Breast [39] dataset which were ranked higher after weighting. The second column indicates if the gene is related to Breast cancer, with the corresponding reference in the third column.

Gene	Cancer-related?	References
S100PBP		
MED8		
DDX20	TRUE	[73]
GPR89A		
ASXL2	TRUE	[74]
PARTICL		
IMP4		
MGAT5	TRUE	[75]
ICOS	TRUE	[76]
AC112220.2		
PHF7		
ATXN7		
DNAJC13		
GAPT		
AC008608.2		
AC106795.2		
SAPCD1		
TNFRSF21	TRUE	[77]
CRYBG1		
MOXD1		
NUP43	TRUE	[78]
CDK13	TRUE	[79]
ZKSCAN1		
CLDN15		
NOM1		
NOL6	TRUE	[80]
ZCCHC7		
DAPK1	TRUE	[81]
MFSD14B		
CIZ1		
BORCS7		
TRIM21	TRUE	[82]
APIP		
FAM111B	TRUE	[83]
SPDYC	TRUE	[84]
AP5B1		
SPTBN2		
P2RY6		
CWC15		
CLEC4A		
AC087239.1		
ETFBKMT		
ZC3H10		
CRYL1		
IFT88		
MIS18BP1		
ZBTB1	TRUE	[85]
ERG28		
PEAK1	TRUE	[86]
RCCD1	TRUE	[87]
TEDC2		
NDE1		
FTO	TRUE	[88]
B3GNT9		
PARD6A		
SNAI3-AS1		
ALOX15		
ULK2	TRUE	[89]
TANC2		
TRIM65	TRUE	[90]
UBE2O		
ZNF532		
ZNF557		
MAP2K7		
ZNF490		
ZNF429		
C19orf47		
IRF2BP1		
AC010331.1		
DNMT3B	TRUE	[91]
PPP1R3D		
SEC14L2		
PMM1		
CRELD2		
ZMAT1		
NKRF		
MMGT1		

bioRxiv preprint doi: https://doi.org/10.1101/2024.11.04.621867; this version posted November 8, 2024. The copyright holder for this preprint (which was not certified by peer review) is the author/funder, who has granted bioRxiv a license to display the preprint in perpetuity. It is made available under aCC-BY 4.0 International license.

Table S6: Small Lengthscale, Subtype Breast Genes Relation to Cancer. This table shows all genes with lengthscale values between 40 to 90 in the Subtype Breast [41] dataset which were ranked higher after weighting. The second column indicates if the gene is related to Breast cancer, with the corresponding reference in the third column.

Gene	Cancer-related?	References
MTFR1L		
PRPF38A		
TBCE		
KIAA1841		
TSN		
CASP8	TRUE	[92]
AC022007.1		
HEMK1		
TRMT10C		
PLD1		
ATP11B	TRUE	[93]
YEATS2		
E2F3	TRUE	[56]
EHMT2	TRUE	[94]
PRRT1		
TREM1	TRUE	[95]
MSC		
SMC2	TRUE	[96]
CIZ1		
GTF3C4		
BRD3OS		
SEC31B		
CD82	TRUE	[97]
BIRC2	TRUE	[98]
UPK2		
HMBS		
GABARAPL1	TRUE	[99]
STAT2		
NUAK1		
RHOF		
SLC7A1		
EFCAB11		
ZNF770		
HOMER2		
POLR2C		
CDYL2	TRUE	[100]
MBTPS1		
CTU2		
DRG2		
BLMH		
RASL10B		
NBR2		
MSI2	TRUE	[101]
QRICH2		
ME2		
CTDP1	TRUE	[102]
DDA1	TRUE	[103]
SLC25A42		
AC006504.5		
STRN4		
TTYH1		
ZNF544		
JAM2	TRUE	[104]
AC245060.5		
NOL12		
GTPBP1		
TAF1	TRUE	[105]
FGF13	TRUE	[106]
LINC00893		

bioRxiv preprint doi: https://doi.org/10.1101/2024.11.04.621867; this version posted November 8, 2024. The copyright holder for this preprint (which was not certified by peer review) is the author/funder, who has granted bioRxiv a license to display the preprint in perpetuity. It is made available under aCC-BY 4.0 International license.

Table S7: Small Lengthscale, Lobular Breast Genes Relation to Cancer. This table shows all genes with lengthscale values between 40 to 90 in the Lobular Breast [40] dataset which were ranked higher after weighting. The second column indicates if the gene is related to Breast cancer, with the corresponding reference in the third column.

Gene	Cancer-related?	References
FBXO2		
IFFO2		
ZNF436	TRUE	[107]
EXTL1		
MTF2	TRUE	[108]
PHTF1		
HAGLR	TRUE	[109]
ORC2		
SATB1	TRUE	[110]
C3orf38		
CMSS1		
CD200	TRUE	[111]
TRIM59	TRUE	[112]
ETV5		
UCHL1	TRUE	[113]
INTS12		
LIFR	TRUE	[114]
ZBED3		
FER	TRUE	[115]
DCP2		
MIR3936HG		
SH3RF2		
SLC36A1		
BNIP1		
HIST1H4J		
NHSL1		
C7orf25		
HUS1		
ZSCAN21		
AASS		
AC004918.1		
FANCG		
NFIL3	TRUE	[116]
AKNA		
PROSER2		
THNSL1		
ENTPD1	TRUE	[117]
CWF19L1		
ARMH3		
ROM1		
AP000438.1		
FDXACB1		
CLEC2D	TRUE	[118]
RASSF3		
PRDM4		
FLT1	TRUE	[119]
BIVM		
ZNF219		
NYNRIN		
CGRRF1	TRUE	[120]
SGPP1		
FAM71D		
GABRB3		
CHST14		
TYRO3	TRUE	[121]
ADAM10	TRUE	[122]
GRAMD2A		
ASPHD1		
PLEKHG4		
PDPR		
MLYCD		
ZC3H18		
CENPBD1		
NLRP1	TRUE	[123]
PIGW		
CEP95		
ABCA5		
MEX3C		
ZNF77		
ZGLP1		
HAUS5	TRUE	[124]
ZNF574		
ZNF628		
ZNF579		
ELMO2		
DIP2A		
PLA2G3		
KCTD17		
APOBEC3C		
TCF20		
JADE3		
TRO		
MECP2	TRUE	[125]

bioRxiv preprint doi: https://doi.org/10.1101/2024.11.04.621867; this version posted November 8, 2024. The copyright holder for this preprint (which was not certified by peer review) is the author/funder, who has granted bioRxiv a license to display the preprint in perpetuity. It is made available under aCC-BY 4.0 International license.

Table S8: Small Lengthscale, Ovarian Genes Relation to Cancer. This table shows all genes with lengthscale values between 40 to 90 in the Ovarian [43] dataset which were ranked higher after weighting. The second column indicates if the gene is related to Ovarian cancer, with the corresponding reference in the third column.

Gene	Cancer-related?	References
HYI		
ECHDC2		
RAVER2		
WDR3		
PAQR6		
DESI2		
STRN		
ACYP2		
PAIP2B		
LIMD1		
PRICKLE2		
BBX		
LSAMP	TRUE	[126]
TMEM39A		
DTX3L		
B3GNT5		
IQCG		
LIN54		
PDE5A		
LARP1B		
HPF1		
DCP2		
WDR55		
CASC15	TRUE	[127]
SLC18B1		
ICA1		
PSPH		
RCC1L		
COG5		
WASL		
KLHDC10		
ZNF775		
INSIG1		
SLC25A6	TRUE	[128]
CXorf36		
HNRNPH2		
PRPS1		
SGK3		
AF117829.1		
ZC3H3		
NPR2		
SCAI		
ZBTB34		
CDK9	TRUE	[129]
LRRC56		
INTS5		
HIKESHI		
CASP1		
SIDT2		
MAP3K8	TRUE	[130]
ERCC6		
FAM149B1		
NOC3L		
LRRC27		
LRRC23		
RDH5		
ARHGEF25		
TXNRD1		
MVK		
B3GLCT		
GCH1	TRUE	[131]
C16orf87		
ZNF319		
SLC12A4		
AC008105.3		
PCYT2	TRUE	[132]
TRAPPC8		
SDCBP2		
BSG		
ZBTB7A		
ZNF443		
ASF1B		
ZNF135		
ATP6V1E1		
ZNRF3		
NOL12		
SREBF2		
